# MicroRNA gatekeepers: Orchestrating rhizospheric dynamics

**DOI:** 10.1111/jipb.13860

**Published:** 2025-02-21

**Authors:** Muhammad Fahad, Leeza Tariq, Wanchang Li, Liang Wu

**Affiliations:** ^1^ Hainan Yazhou Bay Seed Laboratory, Hainan Institute Zhejiang University Sanya 572000 China; ^2^ Zhejiang Provincial Key Laboratory of Crop Genetic Resources, College of Agriculture and Biotechnology Zhejiang University Hangzhou 310058 China; ^3^ National Key Laboratory for Rice Biology, Institute of Biotechnology Zhejiang University Hangzhou 310058 China; ^4^ Institute of Virology and Biotechnology Zhejiang Academy of Agricultural Sciences Hangzhou 310021 China

**Keywords:** biotic and abiotic stress, heavy metals, microbiome, miRNAs, nutrients, rhizosphere

## Abstract

The rhizosphere plays a crucial role in plant growth and resilience to biotic and abiotic stresses, highlighting the complex communication between plants and their dynamic rhizosphere environment. Plants produce a wide range of signaling molecules that facilitate communication with various rhizosphere factors, yet our understanding of these mechanisms remains elusive. In addition to protein‐coding genes, increasing evidence underscores the critical role of microRNAs (miRNAs), a class of non‐coding single‐stranded RNA molecules, in regulating plant growth, development, and responses to rhizosphere stresses under diverse biotic and abiotic factors. In this review, we explore the crosstalk between miRNAs and their target mRNAs, which influence the development of key plant structures shaped by the belowground environment. Moving forward, more focused studies are needed to clarify the functions and expression patterns of miRNAs, to uncover the common regulatory mechanisms that mediate plant tolerance to rhizosphere dynamics. Beyond that, we propose that using artificial miRNAs and manipulating the expression of miRNAs and their targets through overexpression or knockout/knockdown approaches could effectively investigate their roles in plant responses to rhizosphere stresses, offering significant potential for advancing crop engineering.

## Graphical abstract

MicroRNAs play a crucial role in plant communication within the rhizosphere, shaping growth, development, and resilience to biotic and abiotic stresses.

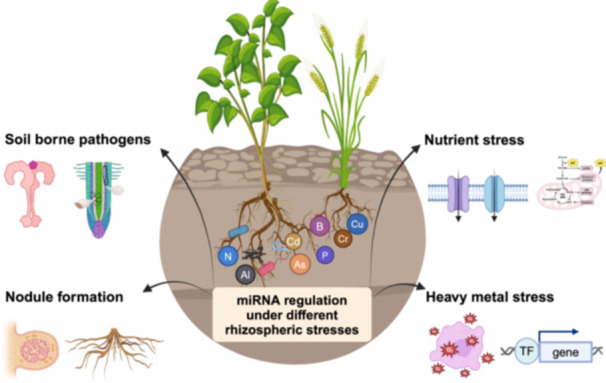

## INTRODUCTION

The rhizosphere is a complex and dynamic environment, consisting of nutrient‐rich soil surrounding plant roots, where plants interact with the diverse community of microbes and respond to various abiotic stresses (Ge et al., 2023). Research shows that these microbial interactions are crucial for plant adaptation, survival, and soil health, particularly under stressors such as nutrient deficiencies, salinity, and heavy metal contamination (Qiao et al., 2021). These interactions influence a wide range of biochemical, physiological, and molecular processes in plants, aiding their resilience to rhizosphere stress. Therefore, this dynamic environment is critical in plant–microbe interactions, nutrient imbalances, and stress responses. Recent studies have revealed that microRNAs (miRNAs) are key regulators in orchestrating these rhizosphere dynamics.

MicroRNAs (miRNAs) are small non‐coding RNAs, typically 20–25 nucleotides in length, that play crucial roles in post‐transcriptional gene regulation across various organisms. The canonical miRNA biogenesis pathway involves two key steps: first, *MIR* genes are transcribed into primary miRNAs (pri‐miRNAs) (Borges and Martienssen, 2015), which are subsequently processed by DICER‐LIKE (DCL) proteins into precursor‐miRNAs (pre‐miRNA), and finally into mature miRNA duplexes. These mature miRNAs are loaded into ARGONAUTE (AGO) proteins, where they typically repress target gene expression through mRNA cleavage or translation inhibition (Borges and Martienssen, 2015; Yu et al., 2017). Interestingly, miRNAs can switch between these two modes of action under specific conditions to enhance their regulatory efficiency (Rong et al., 2024). Additionally, miRNAs can regulate targets epigenetically by triggering DNA methylation, indicating their versatile regulatory mechanisms (Wu et al., 2009, 2010). To date, miRNAs have been shown to play critical roles in plant development and stress resilience, orchestrating responses to both biotic and abiotic stresses (Sajid et al., 2024).

In the rhizosphere, miRNAs play crucial roles in plant–microbe communication and responses to abiotic stress stimuli. For instance, in biotic interactions, miR393, which is activated by pathogen‐associated molecular patterns (PAMPs), regulates plant immunity by modulating auxin signaling through the cleavage of *TRANSPORT INHIBITOR RESPONSE 1* (*GhTIR1*) (Shi et al., 2022). Similarly, the rhizosphere is also critical in mediating plant responses to abiotic stresses, such as nutrient imbalances, salt stress, and heavy metal contamination in soil. Nutrient imbalances, whether due to deficiencies or excesses, severely restrict plant growth and development, posing a growing environmental challenge (Zhao et al., 2023). Furthermore, heavy metal (HM) stress in the rhizosphere significantly reduces plant yields and poses a serious threat at higher concentrations. It negatively impacts plant growth, development, and productivity by altering physiological and metabolic processes (Luo et al., 2022). In response to these challenges, miRNAs have been shown to regulate gene expression in plants under biotic and abiotic stresses, and function both transcriptionally and post‐transcriptionally by forming base pairs with their target mRNAs (Song et al., 2019). Therefore, gaining a profound understanding of miRNAs in rhizosphere dynamics is essential for deciphering the complex relationships between plants and the biotic and abiotic cues they encounter.

In this study, we present a comprehensive overview of miRNAs that respond to various rhizosphere signals, highlighting their expression in a miRNA‐, stress‐, tissue‐, and genotype‐dependent manner. We then explore how miRNAs function as molecular gatekeepers, influencing plant responses to pathogenic microbial stimuli and abiotic challenges such as salt stress, heavy metal contamination, and nutrient availability in the rhizosphere. Moreover, we investigate how miRNAs integrate diverse rhizosphere signals to fine tune plant responses and adaptation mechanisms. Finally, we emphasize that understanding the cell biology of miRNAs within rhizosphere dynamics is essential for unlocking the full potential of these fascinating molecules in future crop engineering advancements.

## MIRNA‐ORCHESTRATED BIOTIC STRESS SIGNALING IN THE RHIZOSPHERE

**Figure 1:**
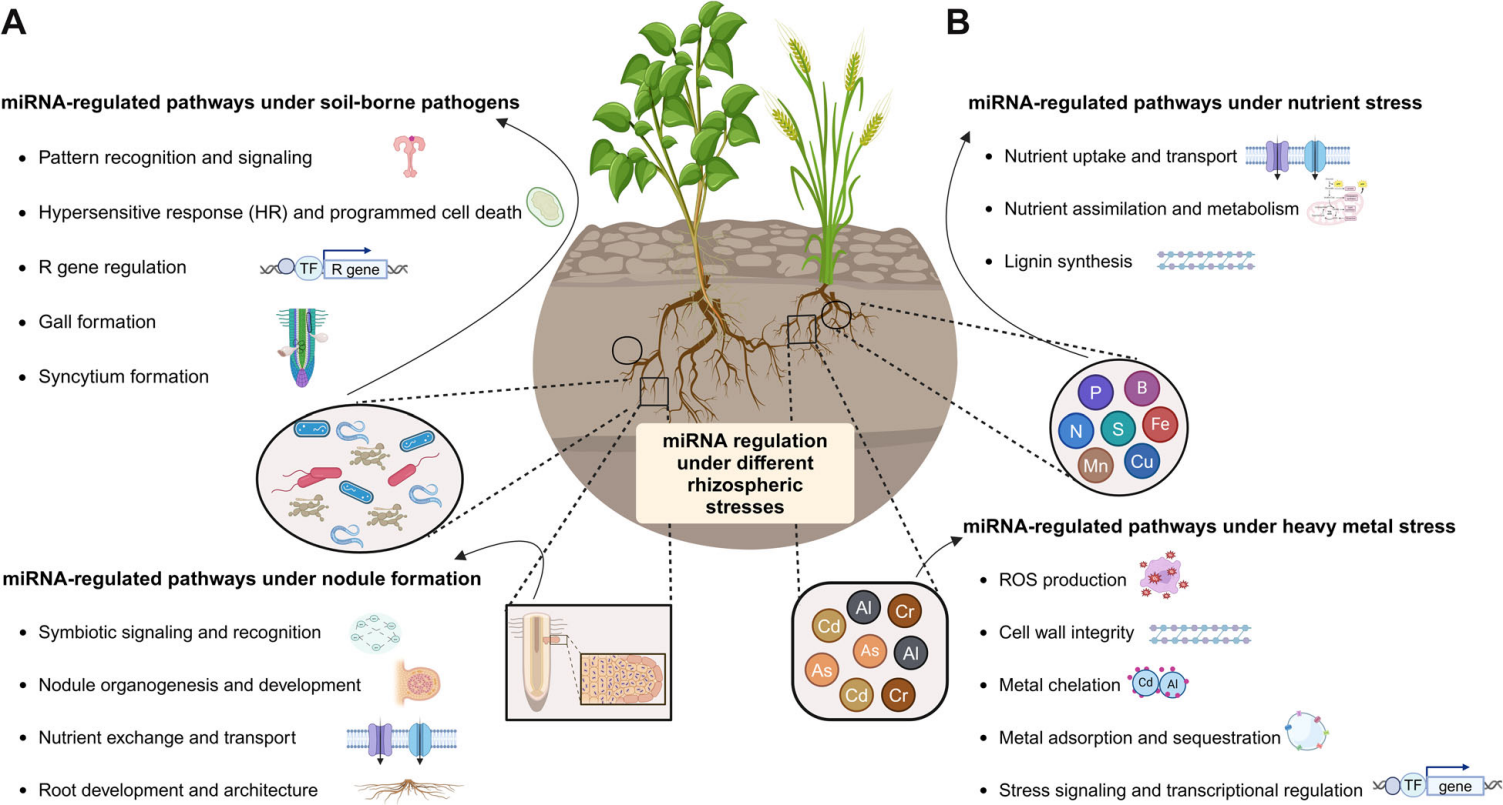
Overview of miRNAs in mediating plant adaptation responses to rhizosphere signaling This figure illustrates the complex network of miRNA‐regulated pathways in plants and their dynamic interactions with diverse rhizosphere signals. **(A)** In response to biotic stresses, such as pathogenic microbes, plants orchestrate the expression of miRNAs that fine tune the regulation of defense‐responsive genes. Specifically, these miRNAs modulate key processes including pattern recognition, cell death, R‐gene expression, and gall formation. During beneficial plant–microbe interactions, miRNAs govern processes such as nodule formation, symbiotic signal perception and transduction, bidirectional nutrient exchange, and root architecture remodeling. **(B)** Under abiotic stresses, such as nutrient imbalances and heavy metal toxicity, miRNAs emerge as crucial regulators. During nutrient stress, they fine tune the expression of genes involved in nutrient uptake, long‐distance transport, assimilation, and metabolic processes. In heavy metal stress, miRNAs control ROS homeostasis, cell wall integrity, metal chelation, and stress signaling. Intriguingly, miRNAs also mediate cross‐kingdom communication, influencing microbial gene expression. This miRNA‐mediated plasticity enables plants to perceive, interpret, and adapt to the complex rhizosphere environment, offering insight for enhancing crop resilience and sustainable agriculture in the face of environmental challenges.

Plant diseases caused by various pathogens have severely affected crop productivity and global economies. The plant's innate immune response to pathogens depends on precise gene expression reprogramming and complex communication with the microbial communities in the rhizosphere (Fahad et al., 2024). miRNAs modulate immune responses at multiple levels, enabling plants to effectively adapt to biotic threats (Figure 1) (Qin et al., 2014). In this section, we discuss the roles of miRNAs in several plant–microbe interactions, including bacteria, root nodules, nematodes, and fungi (Table S1).

### miRNAs crosstalk in the plant–bacteria interactions

Pathogenic bacteria in the rhizosphere can dramatically reduce plant productivity, but plants have evolved various defense mechanisms to counteract these threats. One of the primary strategies involves the regulation of miRNAs, which modulate stress responses and enhance plant immunity against bacterial pathogens (Robert‐Seilaniantz et al., 2011). Extensive research has revealed that miRNAs play multifaceted roles in regulating plant immunity and influencing bacterial responses.

Among the various miRNA families, the miR169 family and its target genes, the NUCLEAR FACTOR Y SUBUNIT‐ALPHA (NF‐YA), are known to regulate biotic stress responses, particularly against bacterial pathogens. For instance, in *Arabidopsis thaliana*, miR169 plays a crucial role in disease resistance to *Ralstonia solanacearum* in CLAVATA1 (CLV1) and CLAVATA2 (CLV2) mutants. These mutants exhibited impaired accumulation of miR169, disrupting the post‐transcriptional regulation of NF‐YA transcription factors. Notably, overexpression of miR169 could restore the enhanced disease resistance, highlighting the complex signaling pathway between miR169 and NF‐YA in plant immune responses (Hanemian et al., 2016). Additionally, when Arabidopsis plants were exposed to sound vibrations in response to *R. solanacearum*, miR397b expression was downregulated. This downregulation activated three laccase genes (*LAC2*, *LAC17*, and *LAC4*), which reinforce cell walls by promoting lignin accumulation, a critical defense mechanism against pathogen invasion (Jung et al., 2020).

Transcriptomic studies have further emphasized the role of miRNAs in plant–bacteria interactions. In ginger infected with *R. solanacearum*, miRNAs such as ppt‐miR1223 and aly‐miR398 were differentially expressed, targeting defense‐related genes like heat shock proteins and NBS‐LRR proteins (Snigdha and Prasath, 2021). Similarly, in tomato, small RNA (sRNA) sequencing identified 69 miRNAs targeting 575 defense‐related genes, uncovering novel miRNA–target modules, such as miR_87‐WRKY and sly‐miR396a‐5p‐GROWTH‐REGULATING FACTORS (GRF), which were further validated by real‐time quantitative polymerase chain reaction (RT‐qPCR) in response to bacterial wilt (Shi et al., 2023). Notably, Sly‐miR482e‐3p was differentially expressed in resistant (“ZRS_7”) and susceptible (“HTY_9”) tomato lines to *R. solanacearum*. The involvement of the endogenous target mimic (eTM482e‐3p‐1), Sly‐miR482e‐3p, and their target NBS‐LRR genes in conferring resistance to bacterial wilt in tomato was confirmed using VIGS technology. Silencing the target mimic (eTM482e‐3p‐1) altered the sensitivity of tomato to the pathogen, highlighting the critical role of the eTM482e‐3p‐1‐Sly‐miR482e‐3p‐NBS‐LRR network in disease resistance (Si et al., 2023). In eggplant, comprehensive sRNA analysis identified 375 miRNAs during *R. solanacearum* infection. Validation through RT‐qPCR revealed complex expression patterns, with miRNAs such as miR414, miR5658, and miR5021 upregulated in susceptible varieties, while others like osa‐miR2873c, exhibited nuanced expression patterns during different infection stages (Kapadia et al., 2023).

While the roles of miRNAs in plant immunity against foliar bacteria are well documented, their impact on bacterial behavior in the rhizosphere, particularly in response to pathogenic bacteria, is an emerging field of study. Recent evidence has suggested that plant miRNAs are not confined to plant tissues but can also move through the rhizosphere and influence bacterial behavior (Cai et al., 2018a; Teng et al., 2018). Specifically, plant‐derived miRNAs have been detected in the rhizospheres of Arabidopsis and *Brachypodium distachyon*, where they modulate gene expression in bacteria like *Variovorax paradoxus*, but not in *Bacillus mycoides*. This finding suggests that plant miRNAs may play a significant role in regulating microbial assembly and plant–bacteria interactions within the rhizosphere (Middleton et al., 2024). By altering bacterial gene expression, miRNAs could influence bacterial behavior and enhance plant defense mechanisms (Venturi and Keel, 2016). These findings highlight the importance of further exploring how miRNAs move from plants to bacteria and their role in shaping the rhizosphere microbiome.

However, while transcriptomic studies provide valuable insight into plant–bacteria interactions, the specific role of plant miRNAs in modulating pathogenic bacterial behavior in the rhizosphere remains understudied. The recent research focused on transcriptomic profiles, but functional studies exploring how miRNAs actively modulate bacterial virulence and community structure are still scarce. A critical gap exists in understanding how miRNAs influence the virulence of pathogenic bacteria and how these interactions contribute to plant disease resistance (Boutet et al., 2022). Given the potential for miRNAs to mediate long‐distance communication between plants and bacteria, it is essential to investigate how plant‐derived miRNAs are transferred to and affect pathogenic bacteria in the rhizosphere (Choi et al., 2017).

Future research could reveal novel mechanisms by which plants defend themselves against microbial threats. Investigating how plant miRNAs influence bacterial gene expression in the rhizosphere may open new avenues for sustainable agricultural practices, disease control, and environmental remediation (Yu et al., 2017). The role of miRNAs in bacteria–plant crosstalk is an emerging field with great potential. However, more foundational research is needed to fully understand how plant miRNAs shape bacterial communities, particularly those of pathogenic bacteria. Gaining a deeper understanding of these interactions is crucial for advancing our knowledge of plant immunity and developing innovative strategies to manipulate microbial communities for crop protection and disease management.

### Bidirectional miRNAs crosstalk in the plant fungus rhizosphere

Fungal pathogens in the rhizosphere pose significant threats to crop sustainability. Advancements in high‐throughput sequencing technology and bioinformatics have highlighted the critical role of host miRNAs in defending against these pathogens, revealing complex molecular communication through various signaling pathways.

**Figure 2:**
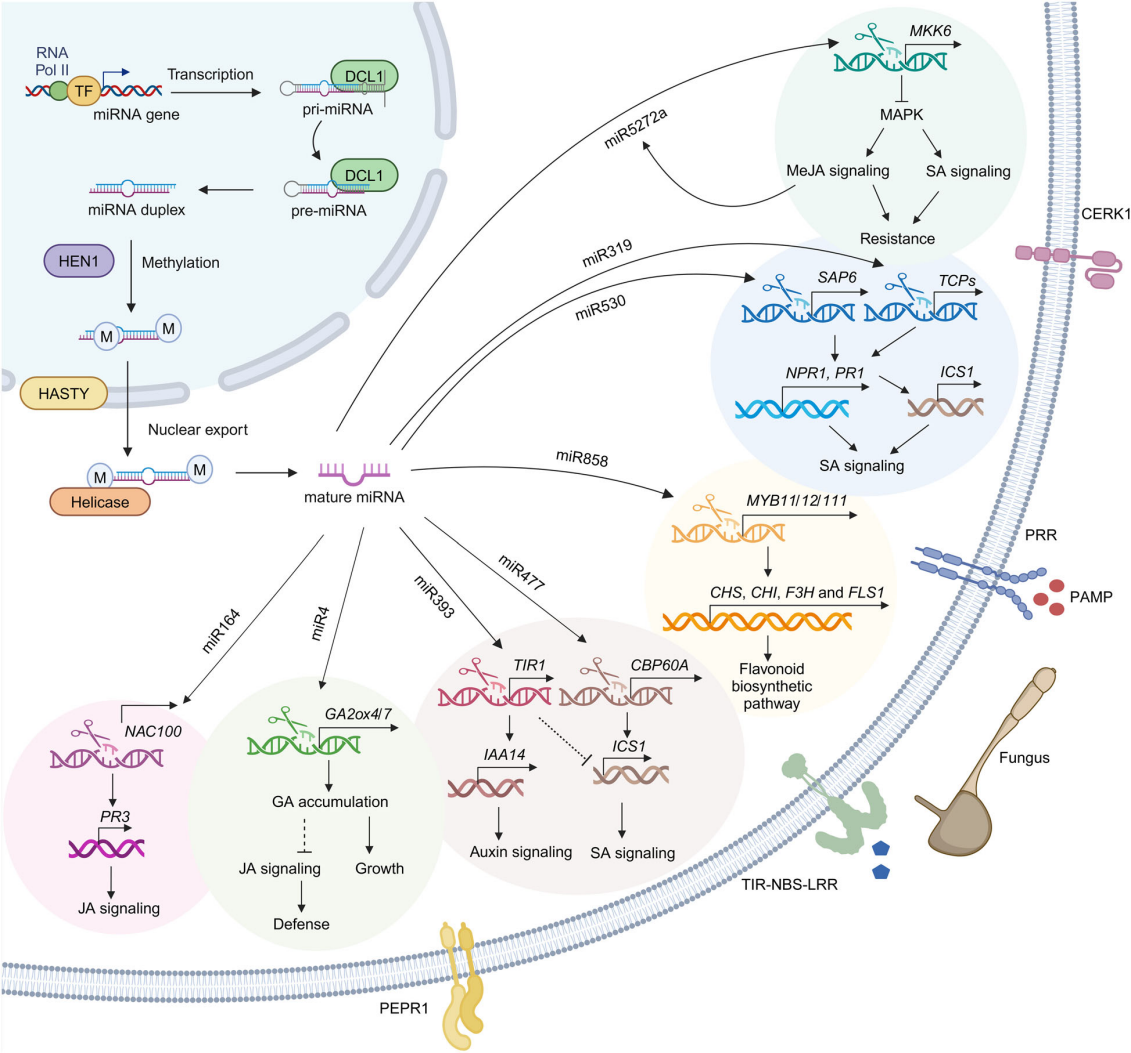
Role of miRNAs in plant–fungus interactions In miRNA biogenesis, HEN1 (Hua Enhancer 1) stabilizes mature miRNAs through methylation, while HASTY (an exportin‐5 homolog) facilitates miRNA export from the nucleus. Helicase aids in unwinding RNA structures during miRNA maturation. miRNAs regulate plant defense against fungal pathogens through various molecular pathways. For example, miR858 negatively affects immunity against *Fusarium oxysporum* by targeting MYB transcription factors (*MYB11*, *MYB12*, and *MYB111*), which modulate ET‐mediated defense responses. In cotton, miR530 targets *GhSAP6* in response to *Verticillium dahliae* infection, enhancing defense, whereas overexpression weakens resistance by downregulating SA‐related genes like *GhNPR1* and *GhPR1*. In contrast, miR477 cleaves *GhCBP60A*, promoting *GhICS1* expression and boosting SA accumulation to strengthen immunity. miR164 targets *GhNAC100*, upregulating the JA‐responsive gene *GhPR3*, thereby enhancing defense against *V. dahliae*. miR393 downregulates *GhTIR1* to influence auxin signaling, while its interaction with *GhIAA14* modulates resistance to *V. dahliae*. Moreover, miR319b fine tunes *GhTCP4*‐like to activate *ICS1* expression via *NPR1*, promoting SA accumulation and increasing resistance to *V. dahliae*. In maize, the *Fusarium verticillioides*‐responsive miRNA, zma‐unmiR4, regulates resistance by targeting *GA2ox4* and *GA2ox7*, with repression of these genes and enhancing susceptibility by disrupting gibberellin signaling and defense‐related gene expression. Finally, miR5272a in cotton modulates defense against *F. oxysporum* by targeting *GhMKK6*, balancing resistance, and preventing excessive hypersensitive response; overexpression of ghr‐miR5272a reduces *GhMKK6* levels and increases susceptibility. These studies highlight the intricate interactions between miRNAs and hormone signaling pathways in regulating plant defense against fungal pathogens.

miRNAs play a crucial role in orchestrating hormonal responses and influencing plant immunity in diverse ways. For instance, miR858 negatively regulates Arabidopsis immunity against *Fusarium oxysporum* by targeting MYB transcription factors (*AtMYB11*, *AtMYB12*, and *AtMYB111*), which are involved in the phenylpropanoid biosynthetic pathway. Interference with miR858 activity using target mimic technology (*MIM858* plants) enhances resistance to fungal pathogens by significantly boosting ethylene (ET)‐mediated defense responses, upregulating defense genes such as *PLANT DEFENSIN 1.2* (*PDF1.2*) and *PATHOGENESIS‐RELATED 4* (*PR4*), and increasing the accumulation of antifungal flavonoids (Figure 2) (Camargo‐Ramírez et al., 2018). A novel *Fusarium verticillioides*‐responsive miRNA, zma‐unmiR4, was identified in maize and shown to regulate resistance through gibberellin (GA) signaling. Its expression is repressed in resistant maize lines and induced in susceptible ones, while its target gene, *GIBBERELLIN 2‐OXIDASE 4* (*ZmGA2ox4*), shows the opposite pattern. Overexpression of zma‐unmiR4 in Arabidopsis enhances growth but reduces resistance, whereas overexpression of *ZmGA2ox4* or its homolog *AtGA2ox7* increases resistance but inhibits growth. Mechanistically, zma‐unmiR4‐mediated suppression of *AtGA2ox7* disrupts bioactive GA accumulation and alters the expression of defense‐related genes during fungal infection. This underscores zma‐unmiR4 as a regulator of the growth–defense balance in response to *F. verticillioides* (Figure 2) (Xu et al., 2022a).

In addition to ethylene and gibberellin, miRNAs modulate salicylic acid (SA), jasmonate (JA), and auxin pathways to enhance plant immunity. For example, the cotton miR530‐*SAP6* module mediates a systemic response to *Verticillium dahliae* infection in roots, where ghr‐miR530 post‐transcriptionally cleaves *SECRETED ASPARTIC PROTEASE 6* (*GhSAP6*) mRNA. Silencing ghr‐miR530 enhances plant defense against *V. dahlia*, while its overexpression weakens plant resistance by downregulating SA‐related gene expressions, such as *NONEXPRESSOR OF PATHOGENESIS‐RELATED 1* (*GhNPR1*) and *GhPR1* (Hu et al., 2023a). The GhmiR477‐*CBP60A* (*CALMODULIN‐BINDING PROTEIN 60*) module regulates the late‐stage SA‐mediated defense response in cotton during *V. dahliae* infection. ghr‐miR477 post‐transcriptionally cleaves *GhCBP60A*, enhancing *ISOCHORISMATE SYNTHASE 1* (*GhICS1*) expression and boosting SA levels to increase cotton immunity (Figure 2) (Hu et al., 2020). In addition, the cotton miR164‐*NAC100* module enhances resistance to *V. dahliae* by cleaving *GhNAC100* (NAM, ATAF, and CUC 100) mRNA post‐transcriptionally via ghr‐miR164. This cleavage prevents *GhNAC100* from repressing the JA‐responsive *GhPR3* gene, boosting plant defense through the JA pathway (Figure 2) (Hu et al., 2020). The cotton miR393‐*TIR1* module regulates defense against *V. dahliae* through auxin signaling. ghr‐miR393 cleaves *TRANSPORT INHIBITOR RESPONSE 1* (*GhTIR1*), and its knockdown increases susceptibility, while overexpression of ghr‐miR393 or *GhTIR1* knockdown enhances resistance. *GhTIR1* interacts with *INDOLE‐3‐ACETIC ACID INDUCIBLE 14* (*GhIAA14*), and the knockdown of *GhIAA14* reduces resistance. Additionally, the simultaneous knockdown of *GhTIR1* and *GhICS1* increases susceptibility. Transcriptome analysis shows that *GhTIR1* knockdown downregulates auxin‐related genes and upregulates SA‐related genes, highlighting the interplay between auxin and SA in cotton defense (Shi et al., 2022). Similarly, a cotton TEOSINTE BRANCHED1/CYCLOIDEA/PCF (TCP4‐like) protein, fine tuned by miR319b, activates *ICS1* expression via *NPR1*, promoting SA accumulation and enhancing resistance to *V. dahliae*. Knockdown of ghr‐miR319b increases resistance, while silencing *GhTCP4*‐like increases susceptibility, as *GhTCP4*‐like activates *ICS1* expression through *NPR1* and promotes SA accumulation, initiating a positive feedback loop that strengthens plant defense against fungal infection (Figure 2) (Jia et al., 2022). These examples highlight the complex interactions between miRNAs and hormone signaling in regulating plant defense against fungal pathogens.

Furthermore, ghr‐miR5272a plays a key role in cotton's defense against *Fusarium oxysporum* by regulating the MAPK kinase gene *MITOGEN‐ACTIVATED PROTEIN KINASE KINASE 6* (*GhMKK6*). Although *GhMKK6* is essential for resistance, its overactivation triggers an excessive hypersensitive response. ghr‐miR5272a helps to regulate this response by targeting the 3′‐untranslated region of *GhMKK6*. Overexpression of ghr‐miR5272a reduces *GhMKK6* levels and disease‐resistance gene expression, making cotton more susceptible to *F. oxysporum*, thus revealing a critical feedback loop in the plant's immune response to fungal pathogens (Figure 2) (Wang et al., 2017). In Arabidopsis, miR773 targets *METHYLTRANSFERASE 2* (*MET2*) to regulate PAMP‐triggered immunity against *F. oxysporum*. Disrupting miR773 activity through target mimics (*MIM773* plants) resulted in the upregulation of *MET2*, which enhanced resistance to the fungal pathogen, while overexpressing miR773 or silencing *MET2* increased susceptibility (Salvador‐Guirao et al., 2018). Therefore, suppressing miR773 could be a promising strategy to improve disease resistance in Arabidopsis. In *Brassica napus*, the miR168‐AGO1 module may facilitate infection by *V. longisporum*. Suppressing miR168 increases AGO1 expression, which enhances fungal colonization. AGO1 knockout mutants exhibited reduced disease symptoms and lowered fungal biomass, whereas wild‐type and miR168b mutants showed greater susceptibility (Shen et al., 2014), highlighting the role of the miR168‐AGO1 module in promoting fungal infection. Moreover, deep sequencing identified 383 miRNAs, with GhmiR395 and GhmiR165 associated with the response to *V. dahlia*. GhmiR165‐REV (REVOLUTA) regulates secondary cell wall formation and vascular patterning, while GhmiR395‐*APS1/3* (*ATP SULFURYLASE 1/3*) modulates sulfur assimilation to combat fungal infection (Mei et al., 2022).

These studies highlight the multifaceted roles of miRNAs in plant immunity across various species, including the regulation of hormone signaling pathways, the modulation of transcription factors, the control of key physiological processes, and the export of miRNAs into pathogens to suppress their virulence genes. Future studies should explore the spatiotemporal regulation of miRNA dynamics in the plant–fungus interactions, and their crosstalk with epigenetic mechanisms, systemic signaling, and metabolic pathways using advanced 'omics approaches. Integrating these studies with evolutionary analyses across species will help to elucidate miRNA‐mediated defense networks, facilitating the development of innovative strategies to enhance crop resistance to fungal pathogens.

### miRNA cues shaping nematode–rhizosphere interactions

Nematodes, among the most abundant and ubiquitous soil animals, significantly influence plant health through rhizosphere interactions in which small‐molecule communication is crucial. Despite the identification of many miRNAs responsive to nematodes, only a few have been thoroughly characterized (Hewezi, 2020).

**Figure 3:**
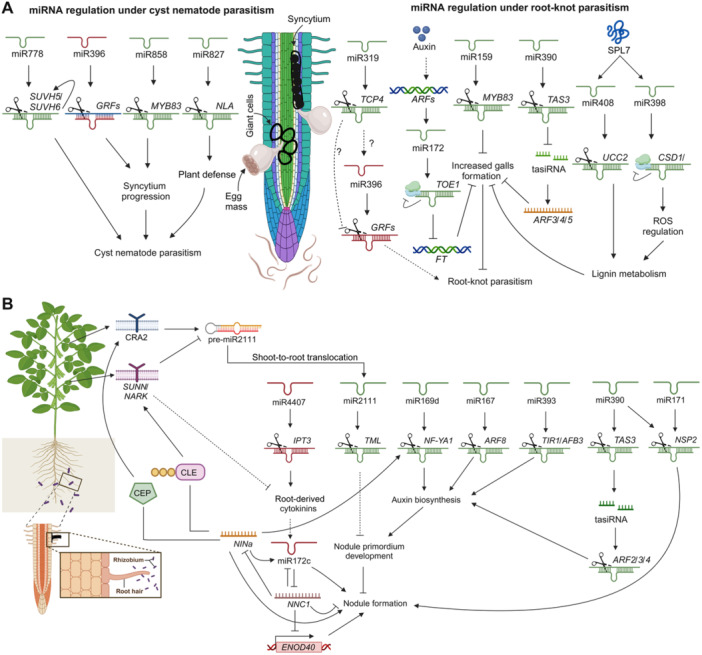
Overview of miRNAs and their target genes in nematode interactions and root nodule symbiosis in plants **(A)** Upregulation of miR408 and miR398, activated by *SPL7* during copper deficiency, is observed in response to RKNs and is localized to nematode‐feeding cells. Knockout mutants reveal the critical role of the *SPL7/MIR408/MIR398* pathway in the development of giant‐feeding cells induced by nematodes. Additionally, miR159 inhibits *MYB33* in galls post‐inoculated with *Meloidogyne incognita*, reducing susceptibility, while miR319 suppresses *TCP4*, enhancing cotton's immune response to RKNs. In tomatoes, differentially expressed miRNAs on Sneb821 and *M. incognita* inoculation revealed that Sly‐miR482d silencing or NBS‐LRR overexpression reduced nematode infection. Modulating miRNA172 expression enhances plant tolerance to RKNs. Furthermore, miR778 regulates *SUVH5* and *SUVH6*, altering root transcriptome, DNA, and histone methylation during *H. schachtii* infection. Induction of miR827 increases susceptibility, while its inactivation upregulates *NLA*, enhancing immunity against nematodes. Last, miR396 targets *GRF1*, confirming its role in RKN infection response. **(B)** In *M. truncatula*, miR169 orchestrates nodule growth and meristem maintenance by targeting *NF‐YA1*. miR2111, transported from shoots to roots in response to rhizobium‐induced CLE peptides, targets the *TML* gene, thereby inhibiting nodule formation. miR167 promotes nodule formation in soybeans by repressing the expression of *ARF8*. Moreover, miR393, associated with auxin perception, influences both determinate and indeterminate nodule formation by regulating TIR1*/AFB3* expression. In addition, miR4407 modulates lateral root emergence and root architecture by suppressing *GmIPT3*, contributing to the AON pathway via miR172c. This miR172c further fine tunes lateral root emergence and root architecture by suppressing *GmIPT3* and is intricately linked with the *GmNINa* pathway, which is crucial for AON. Red color indicates downregulation of miRNAs, while green color indicates upregulation.

Several miRNAs post‐transcriptionally regulate transcription factors during nematode stress, modulating gene expression to enhance plant defense and stress responses. For instance, a study demonstrated that miR408 and miR398, regulated by the *SPL7* transcription factor under copper‐deficient conditions, were upregulated in Arabidopsis and tomato roots infested by root‐knot nematodes (RKNs). These miRNAs localized to nematode‐feeding cells, influencing the development of giant‐feeding cells essential for parasitism. Infection assays in *spl7* and *mir408* knockout mutants, along with miR398‐resistant lines, confirmed the critical role of the *SPL7*/miR408/miR398 module in nematode infestation and feeding site formation (Figure 3A) (Noureddine et al., 2022). Moreover, in Arabidopsis, *miR159abc* mutants exhibited reduced susceptibility to *Meloidogyne incognita*, highlighting the role of miR159 in nematode resistance. However, the upregulation of miR159 in galls, 14 days after inoculation, inhibited *MYB33* expression, suggesting that miR159 may play a role in gall development by inhibiting MYB33 translation (Medina et al., 2017). Similarly, miR858 targets the MYB transcription factor *MYB83* in Arabidopsis, reprogramming the transcriptome during *Heterodera schachtii* parasitism. Overexpression of miR858 reduces susceptibility to nematodes, while *MYB83* overexpression increases susceptibility. RNA‐seq revealed MYB83′s role in hormone signaling and defense, with miR858 regulating MYB83 through a feedback loop during syncytium formation (Piya et al., 2017). Thus, miRNA‐regulated transcription factors are crucial for fine tuning cellular metabolism and physiology by controlling various downstream targets. They often operate in feedback loops to regulate gene expression, though antagonistic interactions with shared targets remain insufficiently studied.

Furthermore, sRNA sequencing libraries from *Meloidogyne javanica*‐infected and uninfected Arabidopsis roots revealed the downregulation of 21‐nt miRNAs and the upregulation of 24‐nt rasiRNAs (repeat‐associated small interfering RNAs) in galls. The miR390/*TAS3* (*TRANS‐ACTING SHORT INTERFERENCE RNA 3*) module, shown to be active in galls, is essential for proper gall formation through AUXIN RESPONSE FACTOR (ARF) regulation, with 24‐nt sRNAs marking gall development (Cabrera et al., 2016). Similarly, during plant–nematode interactions, the miRNA172/*TOE1*/*FT* regulatory model in Arabidopsis suggests that altered auxin signaling, triggered by *Meloidogyne* spp., promotes the accumulation of miRNA172c/d via ARFs. miRNA172 then targets *TARGET OF EARLY ACTIVATION TAGGED 1* (*TOE1*), leading to a reduction of *TOE1* expression and an increase in FLOWERING LOCUS T (FT) transcript levels. *TOE1* likely regulates FT either directly or through unknown intermediaries (Figure 3A) (Díaz‐Manzano et al., 2018). In tomato, sRNA sequencing identified 78 miRNAs that were differentially expressed in plants inoculated with both Sneb821 and *M. incognita*. Silencing Sly‐miR482d or overexpressing its target, NBS‐LRR (*Solyc05g009750*), reduced nematode infection and activated plant defenses. Sneb821 probably inhibits Sly‐miR482d, enhancing tomato immune responses against *M. incognita* (Yang et al., 2022a). These findings demonstrate how miRNAs regulate various aspects of plant–nematode interactions, from gall development to immune responses, by modulating key genes in Arabidopsis and tomato.

Studies have shown that miR319 is one of the most highly upregulated miRNAs following RKN infection. miR319 targets *TCP4* to mediate systemic defense against RKNs by regulating JA synthesis (Figure 3A) (Zhao et al., 2015). Similarly, miR396a downregulates GRF genes to control cell proliferation in roots. In soybean, miR396 downregulation promotes GRF expression to support syncytium formation during the initial stage of infection. Later, the upregulation of miR396 silences GRF genes in the maintenance phase, ensuring proper nematode development in adult females during *Heterodera glycines* infection (Noon et al., 2019). Together, these miRNAs coordinate a balance between defense and growth, contributing to the plant's response to RKN infection.

In Arabidopsis, miR778 downregulates H3K9 methyltransferases SUVH5 (H3K9 methyltransferases SU(var)3‐9 homolog 5) and SUVH6 at nematode‐feeding sites during *H. schachtii* infection. This leads to reduced H3K9me2 deposition and DNA methylation in protein‐coding and miRNA genes, thereby altering gene expression and reprogramming the transcriptome of syncytial cells. Overexpression lines of miR778 and mutants of SUVH5 and SUVH6 exhibited significantly increased susceptibility to *H. schachtii* compared with control plants (Bennett et al., 2022). In another study, inactivating miR827 led to the upregulation of its target gene *NITROGEN LIMITATION ADAPTATION* (*NLA*), resulting in enhanced immunity against the nematode (Figure 3A) (Hewezi et al., 2016). Moreover, sRNA sequencing of soybean cultivars HPZ and W82, which differ in soybean cyst nematode (SCN) resistance, revealed differential expression of 14 known and 26 novel miRNAs in response to SCN infection. qRT‐PCR confirmed distinct expression patterns of specific miRNAs, including novel_miR_106, gma‐miR408a, and gma‐miR3522, in susceptible and resistant cultivars during early SCN infection stages (Lei et al., 2019). Additionally, a novel miRNA, sly_miRNA996, was identified in tomato roots infected by RKNs, exhibiting an anticorrelated expression with its target MYB‐like transcription factor gene (Kaur et al., 2017).

Together, nematode‐activated miRNAs in the syncytium play crucial roles in regulating plant–nematode interactions by modulating defense responses, cell proliferation, and feeding site formation through targeted regulation of transcription factors, E3 ligases, auxin signaling factors, disease‐resistance proteins, growth factors and epigenetic regulators, particularly histone‐modifying enzymes. Future studies, utilizing advanced 'omics approaches and cross‐species comparisons to explore the evolutionary conservation and diversification of miRNA‐mediated defense mechanisms, will help to develop broad‐spectrum nematode resistance in key crops.

### miRNA coordinators of root nodule organogenesis

Root nodule formation begins when compatible rhizobia infect the root, triggering a cascade of developmental processes that leads to nodule initiation, development, and maturation (Ledermann et al., 2021). This process is tightly regulated by the interplay of various plant hormones, particularly auxin, and cytokinin, which significantly influence rhizobium infection and nodule formation (Lin et al., 2020). Plant miRNAs, such as miR160 and miR167, play pivotal roles in the early stages of nodulation by modulating hormone sensitivity (e.g., auxin and cytokinin) and transcription factor expression, which influences nodule development (Nizampatnam et al., 2015; Wang et al., 2015). In the later stages of root nodule development, miRNAs, such as miR172 and miR2111, along with others, further regulate the growth and maturation of the nodules by modulating the Autoregulation of Nodulation (AON) pathway and the responses to environmental signals such as nutrient availability (Tsikou et al., 2018; Wang et al., 2019a, 2019b). Similarly, miR169d regulates the nodule meristematic zone and the differentiation of nodule cells in *M. truncatula* by targeting the transcript that encodes the NF‐YA1 transcription factor (Figure 3B) (Combier et al., 2006; Zanetti et al., 2020).

miR160 regulates soybean nodulation by enhancing cytokinin sensitivity in the early stages of nodule development and increasing auxin sensitivity during nodule maturation (Nizampatnam et al., 2015). In contrast, miR167 positively modulates nodule formation in soybean by repressing the expression of *ARF8* (Wang et al., 2015). Additionally, miR393, associated with auxin perception, plays a significant role in both determinate and indeterminate nodule formation. Overexpressing a miR393‐resistant variant of TIR1, an auxin receptor, *TIR1/AFB3*, or deactivating miR393 with a short tandem target mimic (STTM), increases the number of infection foci and nodules in soybeans (Cai et al., 2017a). Furthermore, miR390 targets the *TAS3* transcript, leading to the production of *trans*‐acting small interfering RNAs that repress *ARF2*, *ARF3*, and *ARF4*. In *M. truncatula*, inactivating the miR390/TAS3 module, by mutating AGO7 or using a target mimic, increases rhizobium infection and nodule formation. However, elevated miR390 levels reduce nodulation organogenesis, rhizobium infection, and the expression of nodulation genes, *NODULATION SIGNALING PATHWAY 1* (*NSP1*) and *NSP2* (Hobecker et al., 2017). Thus, miRNAs regulate nodule formation and maturation by modulating sensitivities to auxin and cytokinin.

The AON model describes that, after compatible rhizobia infection, root‐derived signals, particularly clavata3/embryo surrounding region (CLE) peptides, are synthesized in the roots and transported to the shoot (Yang et al., 2022b). In the shoot, these CLE peptides are perceived by LEUCINE‐RICH‐REPEAT RECEPTOR KINASE (LRR‐RK), including *NODULE AUTOREGULATION RECEPTOR KINASE* (*GmNARK*), *PvNARK*, *HYPER NODULATION ABERRANT ROOT FORMATION* (*LjHAR1*), *PISUM SATIVUM SYM29* (*PsSYM29*), and *SUPERNUMERIC NODULE 1* (*MtSUNN1*), with different receptor kinases involved in different species (Krusell et al., 2002; Searle et al., 2003; Schnabel et al., 2005). CLE peptide perception induces the biosynthesis of miR2111, which is subsequently translocated to the root, where it represses *TOO MUCH LOVE* (*TML*) expression (Tsikou et al., 2018). *TML* encodes a nucleus‐localized Kelch‐repeat‐containing F‐box protein, a component of an E3 ubiquitin complex that recognizes specific substrates via the Kelch‐repeat domain to facilitate their ubiquitination and proteasomal degradation (Takahara et al., 2013). C‐terminally encoded peptides (CEPs) are small peptides that promote rhizobium infection and nodule formation in *M. truncatula*, and their expression is tightly regulated by nitrogen deficiency (Imin et al., 2013; Mohd‐Radzman et al., 2015). Specifically, *MtCEP7* expression is rapidly induced by rhizobia, Nod factors (NF), or cytokinin treatment. *NODULE INCEPTION* (*MtNIN*) directly activates the expression of both *MtCEP7* and *MtCLE13*, suggesting that the cytokinin–*MtCRE1–MtNIN* module coordinates CEP and CLE signaling to fine tune nodule numbers (Laffont et al., 2019). While AON inhibits nodulation, CEPs promote infection through their interaction with the shoot receptor COMPACT ROOT ARCHITECTURE 2 (CRA2). This interaction induces the accumulation of miR2111, which, along with *TML*, acts as a downstream regulatory factor in both the SUNN‐mediated negative and CRA2‐mediated positive regulatory pathways (Huault et al., 2014; Gautrat et al., 2020). These pathways independently regulate nodulation, balancing optimal nodule numbers under varying rhizosphere conditions.

Recent studies in soybean have expanded our understanding of miRNA‐mediated regulation in the AON pathway, revealing the *GmNINa*‐miR172c‐*NNC1* module as a crucial mechanism controlling nodulation. miR172c targets *NODULE NUMBER CONTROL 1* (*NNC1*), relieving its repression of rhizobium‐induced CLE peptide genes (*GmRIC1* and *GmRIC2*), thereby activating the AON pathway. Meanwhile, the *NNC1* interacts with NIN to suppress the expression of *GmRIC1* and *GmRIC2*, providing feedback regulation on nodule formation (Wang et al., 2019a). However, *NNC1* also downregulates miR172c, creating a feedback loop, whereas *GmNINa* relieves *NNC1* repression, promoting miR172c expression and the AON pathway. Hence, *NNC1* and NIN regulate miR172 transcription in opposite ways, suggesting a dynamic regulatory circuit for controlling nodulation (Wang et al., 2019a). During rhizobia presence, LysM Nod Factor Receptors (NFRs) detect NFs, which activate miR172c. miR172c then cleaves *NNC1* mRNAs, reducing *NNC1* levels and enabling *EARLY NODULIN 40* (*ENOD40*) expression to trigger nodule organogenesis (Wang et al., 2014). Another miRNA, miR4407, identified in soybean roots and nodules, regulates lateral root emergence and nodulation by repressing *ISOPENTENYL TRANSFERASE 3* (*GmIPT3*). miR4407 is downregulated upon rhizobium inoculation, leading to reduced nodule numbers, whereas overexpressing *GmIPT3* or a miR4407‐resistant mutant increases nodulation. miR4407 also triggers AON by targeting miR172c and de‐repressing *GmNNC1*, with effects mimicked by exogenous CK (Figure 3B) (Fan et al., 2023). Further temporal and cell‐specific studies are crucial to fully elucidate the miRNA‐mediated regulatory mechanisms in AON and determine their conservation across different legume species.

In addition, miR399 has emerged as a key regulator of nodulation and root growth in response to N and Pi levels. Under N sufficiency, miR399 is regulated by the CRA2 pathway and modulated by the MtCLE35 and MtCEP1 peptides. miR399 accumulation varies with nutrient conditions, influencing root architecture in different ways. At high Pi, overexpression of miR399 reduces nodule number and root biomass, whereas, at low Pi, it primarily affects nodulation by regulating its target gene, *PHOSPHATE 2* (*PHO2*), which is crucial for optimal nodulation (Huertas et al., 2023; Argirò et al., 2024). The evolutionary conservation of the miR399/*PHO2* module plays a central role in coordinating systemic signaling pathways that integrate symbiotic nodulation, root development, and plant N and Pi nutrition. Moreover, miR171h, induced by cytokinin, suppresses *NSP2* expression, a critical transcription factor for nodule development in legumes, thereby reducing nodulation (Hofferek et al., 2014). These studies highlight the diverse miRNA‐mediated regulatory mechanisms governing nodulation in legumes, enabling plants to thrive in N‐deficient conditions. Understanding legume–rhizobium symbiosis, combined with advanced 'omics technologies, offers the potential to transfer nitrogen‐fixing ability to non‐symbiotic crops. Such advancements could significantly reduce or even eliminate the need for N fertilizers, promoting more sustainable agricultural practices.

Taken together, these studies suggest that various plant species may use a conserved immunity mechanism, with miRNAs playing a key role in plant–pathogen interactions (Wu et al., 2017) by modulating PTI and ETI pathways. miRNAs target transcription factors, disease‐resistance proteins, E3 ligases, auxin signaling factors, and growth factors that influence immune responses in the rhizosphere environment. Most miRNAs, such as the miR482/2118 family, regulate NBS‐LRR genes, which play a crucial role in plant disease resistance (Canto‐Pastor et al., 2019; Zhang et al., 2022d). However, to fully understand the mechanisms behind miRNA responses to rhizosphere pathogens, it is essential to investigate their specific functions in this environment. While computational predictions of miRNA targets have been explored, experimental validation is still necessary to confirm these findings. Additionally, the role of miRNAs in the interaction between plant pathogens and nutrient imbalances in the rhizosphere is not yet well understood. miRNA‐based strategies offer promising solutions for combating pathogens while maintaining nutrient homeostasis in crops. A deeper understanding of how miRNAs regulate nutrient dynamics in response to rhizosphere pathogens is vital for improving agricultural practices and enhancing crop resilience, particularly under changing environmental conditions. Discovering new miRNA–target modules, along with advanced genome editing tools, will place miRNAs at the forefront of future crop resistance breeding strategies.

## miRNA‐MEDIATED ABIOTIC STRESS RESPONSES IN THE RHIZOSPHERE

Abiotic stresses in plants, such as heavy metal exposure and nutrient availability, provide dynamic environmental signals that influence plant growth and development. In response, plants have developed intricate strategies to cope with these varying stresses in natural habitats (Tufail et al., 2022). Over the past decade, advanced technologies like next‐generation sequencing (NGS) have facilitated the identification and characterization of miRNAs across various plant species, revealing their distinct functional roles in plant stress tolerance.

### Rhizosphere miRNA heavy metal crosstalk

Soil heavy metal contamination, exacerbated by industrialization and the overuse of agrochemicals, harms plants by inhibiting antioxidant enzymes, disrupting protein expression, and impairing photosynthetic processes. These effects led to stunted root growth and premature leaf senescence. In response, plants utilize miRNA networks to regulate root development, hormone signaling, antioxidant functions, transcription factors, and metal transporter expression to mitigate heavy metal stress (Figure 1) (Sajid et al., 2024). This section reviews current studies on the roles of miRNAs in plant stress responses to heavy metals such as cadmium (Cd), aluminum (Al), arsenic (As), and chromium (Cr) (Table S2).

#### miRNA reprogramming in Cd stress signaling

Cadmium (Cd) is a toxic heavy metal that negatively impacts plant growth, crop yield, and food safety by inducing oxidative stress and disrupting ion balance. Understanding plant responses to Cd stress at the molecular level is crucial to improving Cd tolerance in crops. Recent advancements in transcriptomics and RNA sequencing have identified Cd‐responsive miRNAs that regulate gene expression, thereby enhancing tolerance to Cd stress.

**Figure 4:**
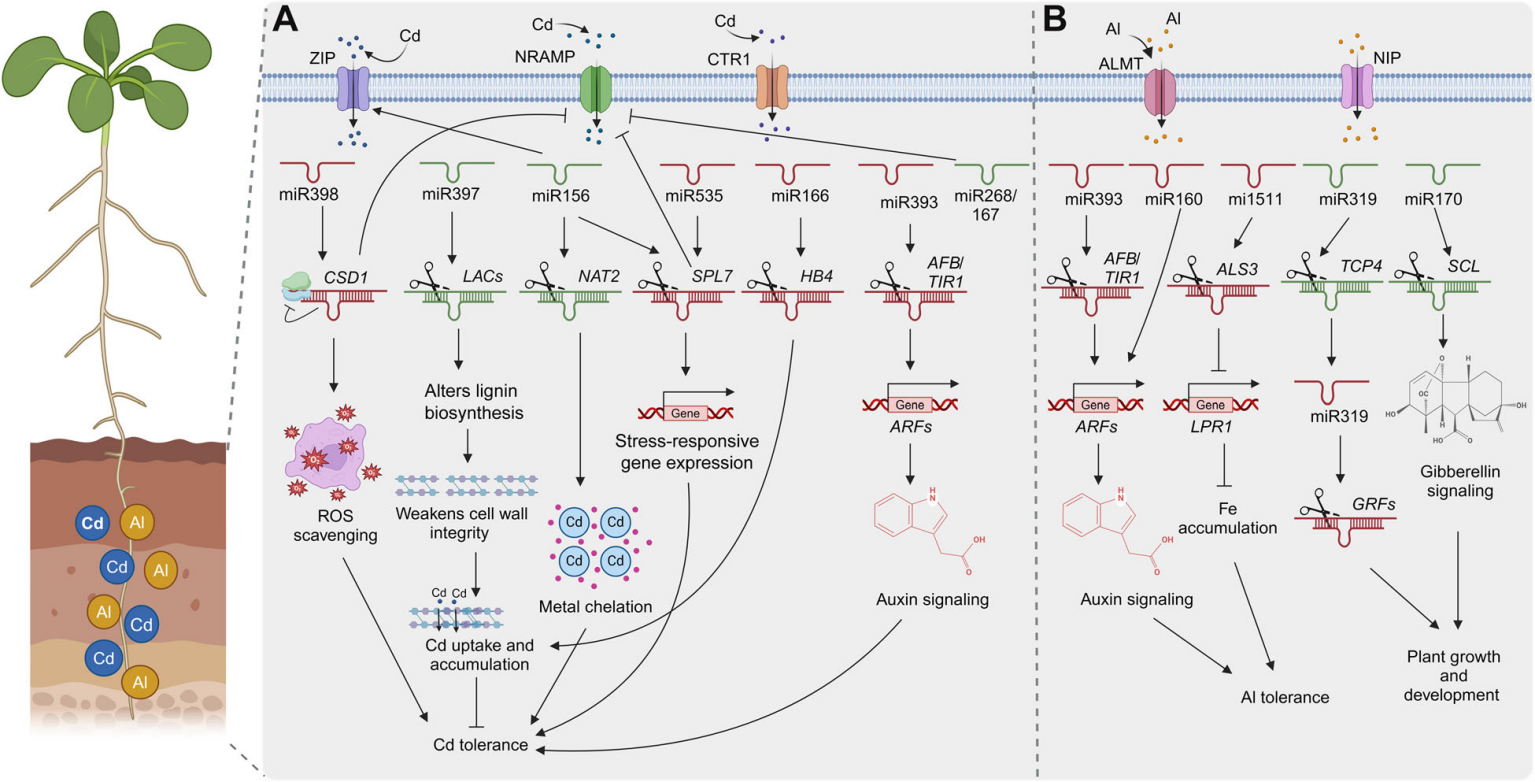
miRNA‐mediated regulation of plant tolerance to cadmium and aluminum toxicity **(A)** In Arabidopsis, the downregulation of miR397 enhances Cd tolerance by increasing the levels of chlorophyll, carotenoids, and lignin, while simultaneously reducing oxidative damage, regulated by lacasses (*LAC2/4/17*). miR398 downregulation under Cd stress leads to the upregulation of its target genes, *CSD1* and *SOD*. In *B. napus*, *BnNRAMP1b*, a target of miR167, contributes to the plant Cd tolerance mechanisms. In rice, miR535 modulates Cd tolerance by regulating *SPL7*, which influences the expression of *NRAMP5*. Overexpressing (OE535) plants show increased Cd sensitivity and accumulation. In barley, miR156g‐3p_3 targets *HvNAT2*, enhancing Cd tolerance and reducing oxidative damage. Furthermore, miR393 targets AFB proteins, enhancing Cd tolerance by modulating auxin signaling pathways. Last, miR166 in rice improves Cd tolerance by regulating the *OsHB4*, reducing oxidative stress, Cd translocation from roots to shoots, and Cd accumulation in grains. **(B)** In barley, the miR393 family regulates auxin sensitivity and Al tolerance by targeting *HvTIR1* and *HvAFB*, with Al exposure suppressing miR393 expression. The miR166b/*HvHOX9* module enhances Al tolerance in barley. In common beans, genetic variation in MIR1511 affects Al tolerance by modulating *ALS3* transcript levels, and miR164 and miR170 regulate gibberellin and auxin signaling pathways for Al stress adaptation. Red color indicates downregulation of miRNAs, while green color indicates upregulation.

miRNAs post‐transcriptionally regulate transporter genes in response to Cd stress, playing a key role in enhancing Cd tolerance in plants. For instance, in rice, elevated miR268 expression under Cd stress downregulates its target gene *NATURAL RESISTANCE‐ASSOCIATED MACROPHAGE PROTEIN 3* (*NRAMP3*). Overexpression of miR268 inhibited seedling growth, increased oxidative damage, and led to higher Cd accumulation, suggesting that miR268 acts as a negative regulator of Cd tolerance via *NRAMP3* suppression (Ding et al., 2017). Similarly, in barley, the Cd‐induced miR156g‐3p_3 targets the *NUCLEOBASE‐ASCORBIC ACID TRANSPORTER 2* (*HvNAT2*) gene, which enhances Cd tolerance and antioxidant capacity. Overexpression of *HvNAT2* increased Cd tolerance and reduced oxidative damage, while RNAi knockdown of *HvNAT2* diminished these effects (Wang et al., 2023a), highlighting its role in stress signaling and the conservation of these regulatory mechanisms. In *B. napus*, 22 NRAMP transporter genes were identified, with *BnNRAMP1b*, induced by Cd stress, functioning as a transporter for Cd, Mn, and Zn. Degradome analysis revealed that miR167 regulates *BnNRAMP1b* at the post‐transcriptional level, emphasizing the importance of miRNA‐mediated regulation in Cd transport (Figure 4A) (Meng et al., 2017). Additionally, overexpression of miR156 (miR156OE) in Arabidopsis reduced Cd accumulation and enhanced tolerance by modulating the expression of Cd uptake and sequestration transporters (AtABCC1). In contrast, decreased levels of miR156 (*MIM156*) resulted in heightened Cd sensitivity and increased expression of the Cd transporter *HEAVY METAL‐ASSOCIATED 4* (*AtHMA4*) in *MIM156* plants (Figure 4A) (Zhang et al., 2020b).

miRNAs primarily modulate plant responses to Cd stress by regulating TFs. In rice, miR535 enhances Cd tolerance by targeting *SPL7*, which negatively regulates *NRAMP5*. This miR535‐*SPL7‐NRAMP5* pathway controls Cd uptake and distribution, with miR535 overexpression reducing Cd accumulation in roots, while its suppression increases Cd sensitivity and accumulation (Yue et al., 2023). A genome‐wide analysis of the tobacco variety TN90 identified 42 SPL genes. miR156 targets multiple SPL genes, including *NtSPL4a*, and is upregulated under Cd stress, showing a significant negative correlation with *NtSPL4a* expression (He et al., 2023). Overexpression of *NtSPL4a* or its miR156‐resistant variant, *NtSPL4aΔ*, in transgenic plants led to earlier flowering, enhanced root development, and reduced Cd content. Transcriptomic analysis revealed that *NtSPL4a* overexpression alters the expression of genes involved in transcriptional regulation, metal transport, and secondary metabolism (Liu et al., 2024a). In rice, miPEP156e, a regulatory peptide from the primary transcript of miR156e, enhances miR156 expression and downregulates SPL target genes. Overexpression or exogenous application of miPEP156e improves Cd tolerance by reducing Cd accumulation and reactive oxygen species (ROS) levels, while its knockout increases Cd sensitivity (Lu et al., 2024). miRNAs targeting SPL transcription factors probably play a key role in plant responses to Cd stress, though the precise regulatory mechanisms require further investigation.

In addition, miRNAs play a significant role in regulating antioxidant defense mechanisms, which are essential for mitigating oxidative stress induced by Cd exposure. In Arabidopsis, downregulation of miR397 increases lignin levels and reduces oxidative damage, which enhances Cd tolerance. This miRNA regulates laccases (*LAC2/4/17*), enzymes involved in lignin biosynthesis, highlighting its role in strengthening the plant's structural defenses against Cd‐induced stress (Ali et al., 2023). Similarly, miR398 is downregulated under Cd stress, leading to the upregulation of its target genes *COPPER/ZINC SUPEROXIDE DISMUTASE* (*CSD1*) and *SUPEROXIDE DISMUTASE 1* (*SOD1*), which encode essential antioxidant enzymes. Overexpression of miR398 in transgenic tomato lines resulted in growth inhibition and increased oxidative damage, suggesting that maintaining a delicate balance in miR398 expression is critical for optimal Cd tolerance (Figure 4A) (Yan et al., 2023a).

In rice, the *HOMEODOMAIN CONTAINING PROTEIN 4* (*OsHB4*) gene, targeted by miR166, negatively affects Cd tolerance and increases Cd accumulation. Overexpression of miR166 downregulates *OsHB4*, improving Cd tolerance by reducing Cd accumulation in the plant (Figure 4A) (Ding et al., 2018). In water spinach, the novel miRNA, IamiR‐4‐3p, showed distinct expression patterns between low‐Cd and high‐Cd cultivars under Cd stress, targeting *GLUTATHIONE S‐TRANSFERASE 3* (*GST3*) and *AWPM19‐like* genes. Overexpression of IamiR‐4‐3p in Arabidopsis reduced *GST3* and *AWPM19‐like* expression, leading to increased Cd accumulation in roots and shoots and compromised apoplastic barriers (Shen et al., 2022).

Advancements in bioinformatics and high‐throughput technologies have significantly improved the identification of miRNAs involved in genotype‐specific responses to Cd exposure. For example, in wheat, genotype‐specific responses to Cd stress revealed differential expression of known and novel miRNAs, along with differentially expressed genes (DEGs), providing insight into adaptation mechanisms (Zhou et al., 2019). In *B. parachinensis*, miR395 targets genes involved in sulfur assimilation in the Cd‐tolerant CX4 cultivar, while miR397, miR393, and miR160 are associated with growth improvement in the low‐Cd‐accumulating cultivar under Cd stress. Other miRNAs are involved in enhancing oxidative resistance in the SJ19 cultivar (Zhou et al., 2017), collectively demonstrating the diversity of miRNA‐mediated responses to Cd stress across different plant cultivars.

In *Brassica* seedlings, Cd treatment induced significant changes in miRNA and mRNA profiles, with distinct expression patterns observed in shoots and roots. Notably, altered expression of *MIR395*, *MIR408*, *MIR397*, *MIR398*, *MIR858*, and several novel miRNA precursors affected key processes such as superoxide detoxification and photosynthesis (Fu et al., 2019). In Cd‐stressed maize roots, specific miRNAs, such as zma‐novel‐miR30996‐3p, zma‐novel‐miR21151‐3p, zma‐novel‐miR18642‐3p, and zma‐miR171k‐5p, target genes involved in ABC transporters, peroxisomes, GSH metabolism, and the ubiquitin–proteasome system. These miRNA–gene interactions contribute to reduced Cd accumulation and enhanced tolerance in the Cd‐tolerant L63 genotype compared with the Cd‐sensitive L42 genotype (Teng et al., 2023). These findings underscore the complex role of miRNAs in regulating gene responses to Cd stress, highlighting their significance in genotype‐specific metal homeostasis and tolerance mechanisms. Ongoing research is unveiling potential strategies for developing Cd‐tolerant crops through miRNA‐mediated gene regulation, offering promising solutions for improving food security in Cd‐contaminated areas.

#### miRNA in Al stress response and tolerance

Plants have developed various detoxification strategies to cope with acidic soils rich in aluminum, such as organic acid efflux, Al redistribution, and Al sequestration within cells (Yan et al., 2023b). To date, several miRNAs have been shown to play key roles in plant root development and response to Al stress (Jiang et al., 2023), indicating a complex miRNA‐mediated communication network in the rhizosphere.

Phytohormone signaling, particularly auxin, is crucial for regulating root growth under Al stress. The miR393 family, which negatively regulates auxin receptors TIR1/AFBs, is involved in auxin‐mediated developmental processes and root growth. Specifically, in barley under Al stress, miR393 targets *HvTIR1* and *HvAFB*, with its expression suppressed in the root apex. Overexpression of miR393 alleviates Al‐induced root damage by modulating root sensitivity to Al via auxin signaling. This is supported by evidence showing increased root sensitivity when miR393 is inhibited and impaired Al‐induced root growth inhibition with exogenous auxin application (Bai et al., 2017). This suggests a mechanism for how plants interpret environmental signals and regulate root growth under Al stress. In barley, the miR166b/*HvHOX9* module plays a crucial role in Al tolerance. *HvHOX9*, a homeobox‐leucine zipper transcription factor, is a target gene of miR166b. Under Al stress, *HvHOX9* is upregulated in the root tips of the Al‐tolerant XZ16 genotype. Silencing *HvHOX9* leads to increased Al accumulation in the root cell wall and reduced H^+^ influx. This enhances Al tolerance by minimizing Al binding in the root cell wall and increasing apoplastic pH, which aids in Al detoxification (Feng et al., 2020).

In addition, the Al‐tolerant barley genotype XZ29 exhibits downregulation of miR319‐mediated *HvTCP4* expression and reduced miR396 expression, which contributes to Al tolerance by alleviating root inhibition and enhancing root cell proliferation, respectively. Other miRNAs, such as miR160, miR393, and PC‐miR1, also play important roles in the Al response in barley (Wu et al., 2018). Similarly, in common beans, microarray analysis identified 28 Al‐responsive miRNAs, with miR393 targeting *TIR1*, miR164 targeting *NAC1*, and miR170 targeting SCARECROW‐like protein (SCL), highlighting their roles as key regulators of gibberellin and auxin signaling, which are essential for adaptation to Al stress (Mendoza‐Soto et al., 2015).

Genetic variation in miRNA‐mediated Al tolerance has also been observed. In common beans, genetic analysis revealed that a deletion in the *MIR1511* gene led to increased expression of its target, *ALUMINUM SENSITIVE 3* (*ALS3*), under Al toxicity. Genotypes lacking miR1511 showed enhanced Al tolerance, while miR1511 overexpression increased Al sensitivity, demonstrating the importance of the miR1511‐*ALS3* module in regulating Al tolerance (Figure 4B) (Ángel Martín‐Rodríguez et al., 2021). In *M. truncatula*, mtr‐miR156g‐3p targets genes that are involved in root cell growth, while three novel miRNAs (novel_miR_36, novel_miR_182, and novel_miR_135) regulate Al‐tolerant transcription factors, contributing to the plant's ability to cope with Al toxicity (Lu et al., 2023).

Genotype‐specific miRNA responses to Al stress have also been observed in other species. In soybean, deep sequencing of miRNA libraries from Al‐tolerant and Al‐sensitive genotypes revealed differential expression patterns of 453 miRNAs. Conserved miRNAs such as gma‐miR396c/k, gma‐miR166k/o, and gma‐miR390g promoted root elongation in the Al‐tolerant genotype BX10, while gma‐miR169r was associated with increased oxidative stress in the Al‐sensitive BD2 genotype (Huang et al., 2018). Furthermore, in olive, sRNA‐seq identified 352 miRNAs, including 196 conserved and 156 novel miRNAs, in the roots of two genotypes (FS and ZL). Comparative analysis revealed 11 miRNAs with significantly different expression patterns between FS and ZL genotypes in response to Al stress (Wu et al., 2023). Differential expression of miRNAs was observed between Al‐sensitive and Al‐tolerant sugarcane cultivars, with 394 miRNAs showing distinct expression patterns, which were validated by real‐time PCR. MapMan™ “BIN” ontology predicted target genes of these miRNAs, including ARF, water stress response *CBL‐INTERACTING PROTEIN KINASE 1*, and cell growth‐related genes such as MYB and LRR proteins (Silva et al., 2019).

In *Vitis quinquangularis*, sRNA‐seq analysis of leaves treated with Al revealed eight differentially expressed miRNAs targeting 161 genes associated with endoplasmic reticulum stability and stress resistance. Overexpression and silencing of miR172b and miR477b‐3p confirmed their roles in plant stress responses by targeting specific mRNAs (Jiang et al., 2023). Similarly, sRNA analysis of flax cultivars with varying Al tolerance identified 97 miRNAs from 18 families, with miR393, miR390, and miR319 showing altered expression patterns after Al treatment, particularly between sensitive and resistant genotypes (Dmitriev et al., 2017), suggesting their role in cultivar‐specific Al stress response. Thus, these findings on miRNA‐mediated adaptation to Al stress in the rhizosphere highlight the remarkable complexity and diversity of these small regulatory molecules. By understanding these miRNA‐mediated pathways, researchers can design targeted strategies to improve crop resilience, potentially leading to better yields in challenging environments.

#### Role of miRNAs in plant adaptation to arsenic stress

Arsenic (As) is a toxic metalloid that poses health risks to both plants and humans by entering the food chain through contaminated crops. It increases ROS production, disrupts photosynthesis, and alters metabolism in plants (Pagano et al., 2021). Therefore, understanding miRNA regulation during As exposure is essential to uncovering the mechanisms that help plants adapt to arsenic stress.

In rice, Osa‐miR156j is significantly downregulated under As stress and targets genes such as lectin receptor‐like kinases and zinc finger proteins. This regulation plays a crucial role in enhancing As stress tolerance by modulating lipid metabolism and stress response pathways (Pandey et al., 2020). In contrast, overexpression of miR528 in rice led to increased sensitivity to As(III), correlating with the upregulation of target genes associated with lignin biosynthesis and Cu homeostasis, and resulting in heightened oxidative stress and altered amino acid content in both roots and leaves (Liu et al., 2015a). In a comparative study of rice genotypes LARG and HARG, exposure to As(V) and As(III) led to the upregulation of miR396, miR399, miR408, and miR528, and the downregulation of miR164, miR171, miR395, and others. These miRNAs target genes involved in sulfur assimilation and oxidative stress management, playing key roles in the As stress response (Sharma et al., 2015).

Additionally, miR408 is involved in the downregulation of GLUTATHIONE *S*‐TRANSFERASE (GSTU25) and sulfur reduction pathway genes, such as ADULT‐PLANT RESISTANCE (APR) and ATP SULFURYLASE (APS), in Arabidopsis under As stress, contributing to the detoxification mechanism (Kumar et al., 2023). In *B. juncea* roots, miRNA expression analysis revealed changes in 69 miRNAs, targeting genes involved in developmental processes, sulfur metabolism, and hormone signaling (Srivastava et al., 2013). Similarly, in maize, deep sequencing identified 57 differentially expressed miRNAs under As(V) stress that regulate genes involved in growth, metabolism, ROS generation, and hormone signaling (Ghosh et al., 2017). Together, these studies highlight the involvement of miRNA‐target gene regulatory modules in crucial processes such as detoxification, uptake, transport, and stress tolerance across species. However, knowledge of miRNA roles during early As exposure remains limited. Therefore, further research into miRNA‐guided genetic mechanisms could pave the way for developing crops with improved As resistance, addressing critical food safety concerns.

#### miRNA‐mediated adaptive mechanisms to chromium stress response in plants

Chromium (Cr), a prevalent industrial pollutant, causes significant phytotoxicity in plants, including suppressed seed germination, disrupted nutritional balance and enzymatic activities, reduced root growth, and oxidative stress (Ao et al., 2022). Despite the importance of understanding Cr stress responses in plants, research on Cr‐responsive miRNAs and their target genes has been limited. Recent high‐throughput sequencing studies have identified diverse miRNAs that regulate plant responses to Cr stress.

For instance, in maize, Cr(VI) stress upregulates miR444f, which targets the ABC transporter G29, potentially increasing Cr accumulation in roots. Additionally, miR159c enhances GAMYB expression, disrupting ROS scavenging and impairing root growth (Adhikari et al., 2023). In *Miscanthus sinensis*, Cr treatment led to differential expression of miR167a, novel_miR15, and novel_miR22 in roots, targeting genes involved in Cr transport. Additionally, miR156a, miR164, miR396d, and novel_miR155 were associated with detoxification processes (Nie et al., 2021). In radish roots under Cr stress, miR156, miR159, miR160, and other conserved miRNAs were downregulated, targeting stress‐related transcription factors and ABC transporter proteins. In contrast, miR161, miR172, miR390, and miR394 were upregulated, enhancing stress response mechanisms (Liu et al., 2015b).

In rice under Cr stress, miR156, miR159, and miR160 were differentially expressed, targeting ABC transporters, transcription factors, and heat shock proteins involved in detoxification. Specifically, osa‐miR159 suppresses MAPK activity and enhances auxin signaling, highlighting its role in balancing these pathways (Dubey et al., 2020). A comparative study of the Cr‐sensitive tobacco genotype Yunyan2 and the Cr‐tolerant genotype Guiyan1 identified 53 conserved and 29 novel miRNA families. The upregulation of 11 miRNA families in Guiyan1 and 17 in Yunyan2 highlights distinct miRNA responses to Cr stress, with miR6149 downregulated only in Yunyan (Bukhari et al., 2015). Genome‐wide studies have identified numerous miRNAs and their targets involved in plant responses to Cr stress, but our understanding of the underlying mechanisms remains incomplete.

In summary, these studies validate the involvement of miRNAs in regulating plant responses to heavy metal stress, and elucidating the mechanisms underlying these miRNA‐mediated regulatory networks could pave the way for enhancing crop tolerance to metal toxicity. However, while numerous miRNAs associated with metal stress have been identified across plant species, the majority remains functionally uncharacterized, leaving their specific roles largely unclear. Previous findings demonstrate that miRNAs are key regulators within intricate metal stress response networks, with their target genes encoding enzymes and transcription factors. Importantly, bioinformatics predictions have uncovered potential miRNA targets that require experimental validation to elucidate the intermediate pathways underlying miRNA‐mediated heavy metal tolerance. The integration of approaches such as reverse genetics, miRNA/degradome sequencing, CRISPR‐Cas9, and STTMs will provide an in‐depth investigation. These techniques will help to unravel the mechanisms by which miRNAs regulate their targets and participate in metal stress regulatory networks. Together, these efforts could pave the way for enhancing crop tolerance to metal toxicity through miRNA‐based strategies.

### miRNA caretakers of rhizospheric nutrient homeostasis

Plants require efficient nutrient utilization and tolerance to deficiencies to thrive in diverse rhizosphere conditions. Certain miRNAs act as crucial regulators in low‐nutrient environments, coordinating post‐transcriptional modifications to maintain nutritional homeostasis. Understanding miRNA‐mediated regulation of nutrient signaling pathways offers insight into improving nutrient utilization efficiency and plant productivity (Figure 1). In this section, we discuss the roles of miRNA in macro and micronutrient regulation in plants (Table S3).

#### Role of miRNAs in nitrogen assimilation and metabolism

Nitrogen (N) is essential for plant growth and acts as a signaling molecule that regulates gene expression and various physiological responses. Both excess and deficiency of N can adversely affect plant development (Zhu et al., 2023). To cope with N fluctuations, plants have developed adaptation mechanisms. Recently, miRNAs have been identified as key regulators of plant N assimilation and metabolism, offering the potential for improving N use efficiency (NUE). These small regulatory molecules are involved in N uptake, transport, and assimilation, helping plants adapt to N deficiency (Zhang et al., 2022b).

Over the past decade, there has been a growing interest in the functional roles of miRNAs in N deficiency responses. For example, miR167 regulates root growth in response to N availability. miR167 specifically targets ARFs, particularly *ARF6*, and *ARF8*, which are crucial for lateral root growth and reproductive organ formation in plants such as Arabidopsis and cotton (Gutierrez et al., 2012). Insights from microarray hybridization of root samples revealed that *ARF8* is expressed in both pericyclic cells and horizontal root caps in Arabidopsis roots treated with nitrate (Gifford et al., 2008; Asim et al., 2020). Under N deficiency, *ARF8* enhances lateral root development and shows high expression in the pericycle cells and lateral root caps. However, the downregulation of miR167 in plants exposed to N stress promotes lateral root growth (Asim et al., 2020; Jagadhesan et al., 2022). N stimulation increases *ARF8* expression and inhibits miR167, leading to the accumulation of *ARF8* accumulation in pericycle cells, as confirmed by GUS fusion and qRT‐PCR analysis (He et al., 2014), suggesting that miR167 plays a critical role in the adaptation of both dicots and monocots to N‐limited conditions.

In addition, miR393 is another important player in N‐responsive root development. miR393 is activated by various N sources, including glycine and ammonium nitrate (Guan, 2017). In Arabidopsis, miR393 targets transcripts of auxin receptors, including *AFB1*, *AFB2*, *AFB3*, and *TIR1*. The miR393‐*AFB3* module is essential for root adaptation to N deprivation, regulating both primary and lateral root growth in response to nitrate (Vidal et al., 2010). Similar mechanisms have been observed in soybean, where miR393d modulates auxin sensitivity and lateral root growth in nodular tissues (Cai et al., 2017a). These findings underscore the importance of miR393 and *AFB3* in nitrate‐induced root structure changes via the auxin signaling pathway.

The role of miRNAs in N metabolism extends beyond root development. For instance, miR444 has emerged as a key modulator of N assimilation and transport in several plant species. In rice, the miR444‐*OsMADS27* module is crucial for root growth and stress adaptation (Pachamuthu et al., 2022). Overexpression of Osa‐miR444a, which targets the MADS‐box transcription factor gene *OsMADS25*, increases N accumulation and N transport‐related gene expression under high N conditions but reduces N transport and adaptability under low N environments (Shin et al., 2018). In wheat, *TaMIR444a* and its tobacco homolog *NtMIR444a* are upregulated under N deprivation, with inverse expression patterns observed in their target genes, suggesting a conserved role for miR444a in mediating plant responses to N deficiency. Overexpression of *TaMIR444a* in tobacco enhanced plant growth, N content, antioxidant enzyme activities, photosynthesis, and biomass under N starvation (Gao et al., 2016). Despite these findings, the specific mechanisms by which miR444 regulates N assimilation and transport remain unclear.

Research shows that N deficiency significantly reduces the expression of NF‐YA genes, including *NF‐YA2*, *NF‐YA5*, and *NF‐YA8*, which are targets of miR169 in Arabidopsis (Liang et al., 2012; Xu et al., 2014). Plants overexpressing miR169a show increased vulnerability to N shortage and absorb less N because NF‐YA inhibits the expression of nitrate transporter genes *AtNRT1.1* and *AtNRT2*. This highlights the intricate regulatory mechanism controlled by miR169 (Liang et al., 2012). In rice, overexpression of miR169o enhances NUE under low N conditions but increases susceptibility to bacterial blight (Yu et al., 2018). Moreover, the specific isoform osa‐miR169a in rice strictly regulates the post‐transcriptional regulation of *OsNF‐YA5* among other NF‐YA members, indicating that the *OsNF‐YA5*/osa*‐*miR169a module is crucial for regulating NUE and adaptation to N deficiency (Seo et al., 2023). Similar mechanisms involving the NF‐YA/miR169 module have been observed in wheat under N‐limited conditions (Qu et al., 2015). These findings indicate that the NF‐YA/miR169a module plays a conserved role in plant N response, although its underlying mechanisms and targets require further investigation.

Studies have demonstrated that NAC transcription factors are post‐transcriptionally regulated by miR164 and play crucial roles in stress responses. In apple, *NAC1*, a target of miR164, regulates the expression of the high‐affinity nitrate transporter *MhNRT2.4* and citric acid transporter *MULTIDRUG AND* TOXIC *COMPOUND EXTRUSION* (*MhMATE*), affecting root N uptake. Silencing *MhNAC1* enhances N uptake and citric acid secretion in roots, improving tolerance to low N conditions, while overexpression of *MhNAC1* or silencing miR164 has the opposite effect (Wang et al., 2024). Additionally, overexpressing sugarcane miR156 in Arabidopsis improves N assimilation, indicating its potential for enhancing NUE in sugarcane (Gao et al., 2022).

Comprehensive studies using high‐throughput sequencing techniques have revealed the complexity of miRNA involvement in N metabolism. In maize, multiple miRNAs, including miR166, miR169, miR528, and miR408, were found to be downregulated under N deficiency (Yang et al., 2019). Similarly, using two genotyping systems, 14 types of miRNA in maize were identified as being susceptible to transient or chronic low N levels (Zhao et al., 2013). In potato, a study identified 119 conserved miRNAs from 41 families and over 1,000 putative novel miRNAs, many of which were differentially expressed in shoots and roots under low N stress (Tiwari et al., 2020). These studies highlight the crucial role of miRNAs in plant development, growth, and adaptation to N fluctuations. While key players have been identified, the complex interactions between miRNAs, target genes, and other regulatory elements remain to be fully understood. Further research will focus on unraveling these relationships to enhance crop improvement, potentially reducing fertilizer use, agricultural costs, and environmental impact.

#### miRNA regulatory circuits in phosphate deficiency response in the rhizosphere

Phosphorus (P) is essential for plant growth and development, yet it often exists in inaccessible forms to plants in the rhizosphere. To address this challenge, plants expand their root systems, enhance high‐affinity Pi uptake, adjust metabolism to maintain intracellular Pi balance, and secrete phosphatases and organic acids to mobilize Pi from organic compounds (Zhao et al., 2023). These mechanisms highlight the significance of miRNAs in orchestrating plant adaptive response to Pi deficiency.

miR399 is a key player in Pi deficiency responses, capable of long‐distance translocation from the shoot to the root as a systemic signal, and executing its function in the roots (Pant et al., 2008; Pei et al., 2024). Under Pi‐limiting conditions, the transcription factor *PHOSPHATE STARVATION RESPONSE 1* (*AtPHR1*) upregulates miR399, which then translocates from shoot to root as a systemic signal, where it targets *PHOSPHATE OVER‐ACCUMULATOR 2* (*PHO2*), an E2 ubiquitin conjugate (Bari et al., 2006). PHO2 normally facilitates the degradation of PHO1, a protein crucial for Pi transport from root cells to aerial parts of the plant (Liu et al., 2012). It also affects the expression of specific Pi transporter genes (PHTs) (Hu et al., 2011). By suppressing PHO2, miR399 enhances Pi acquisition and root‐to‐shoot translocation (Liu et al., 2014).

Similarly, in cucumber, miR399 was initially detected in the major veins and petiole vascular bundles of older source leaves; however, its accumulation was observed in the phloem sap upon exposure to Pi starvation (Zhang et al., 2016b). This miR399‐mediated systemic regulation is crucial, as the miR399‐*PHO2* regulatory module is evolutionarily conserved across various plant species (Bari et al., 2006; Cuperus et al., 2011; Lin et al., 2018). A recent study using Arabidopsis mutants revealed that mature miR399 moves independently of its sequence, biogenesis, and length. The miR399/miR399* duplex acts as a mobile entity, silencing *PHO2* expression in root tissues via the root phloem pore pericycle, thereby coordinating shoot Pi demand with its acquisition and translocation (Chiang et al., 2023).

**Figure 5:**
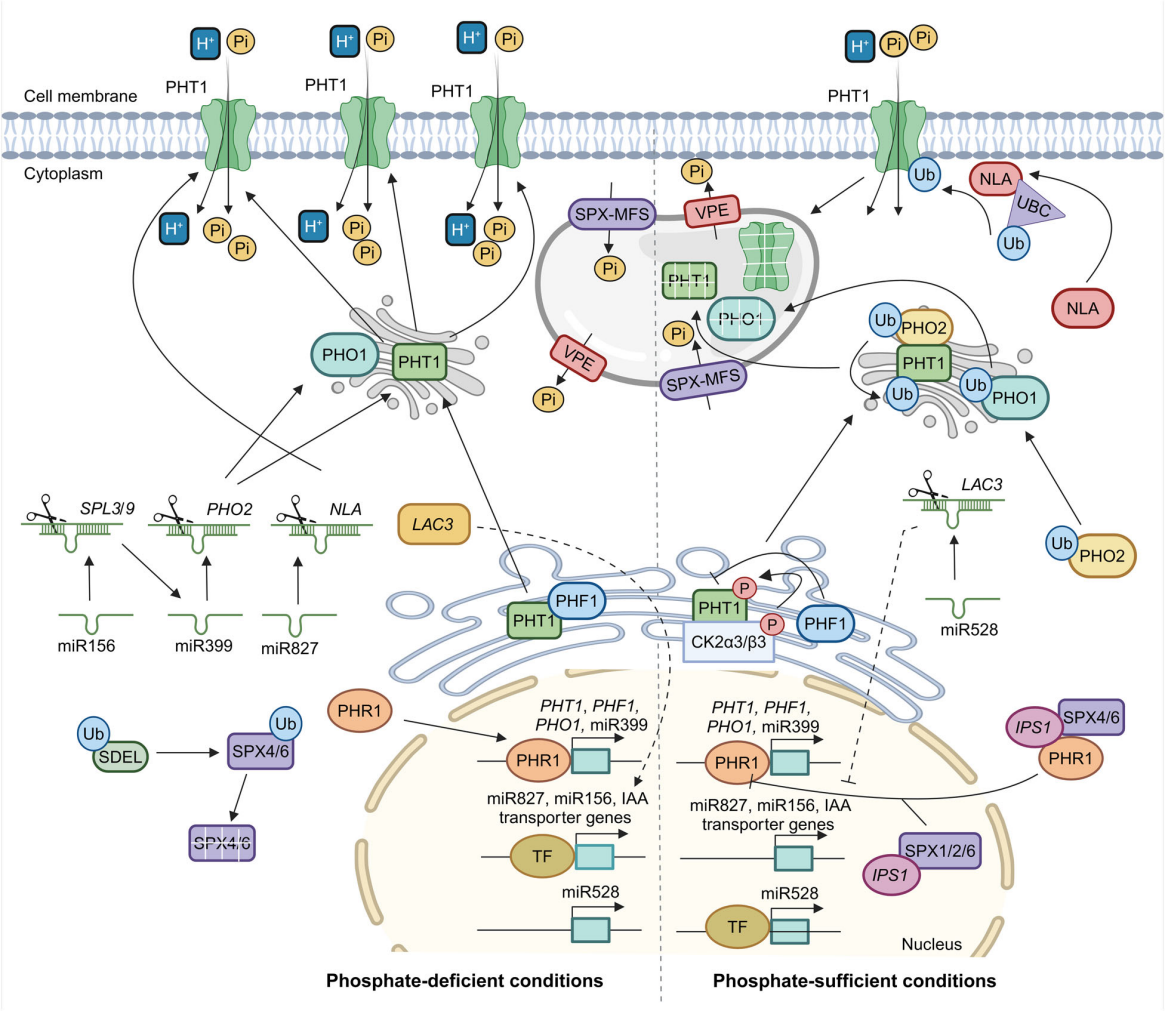
miRNA‐mediated regulation in phosphate homeostasis miR399, a critical player in Pi deficiency responses, targets E2 ubiquitin‐conjugating *phosphate over‐accumulator 2* (*PHO2*), to regulate Pi acquisition and root‐to‐shoot translocation. This regulation is orchestrated by the transcription factor *At‐PHR1*, which activates miR399 expression under Pi‐limiting conditions. Additionally, miR399 moves a long distance from shoots to roots, playing a crucial role in phosphate homeostasis across various plant species. In a similar manner, miR827 translocates between roots and shoots during Pi starvation, influencing phosphate transport and storage by targeting distinct SPX domain‐containing genes in Arabidopsis and rice. In maize, miR528 negatively regulates phosphate uptake, and its suppression enhances tolerance to low‐Pi environments by upregulating Pi absorption‐related genes. These processes are further complicated by stress‐responsive regulatory networks involving TFs and miRNAs that fine tune phosphate homeostasis. TFs such as *OsMYB2P‐1* and *OsWRKY74* in rice regulate the expression of the osa‐miR399 family members under phosphate deficiency. Moreover, miR156, along with its target *SPL3* and *SPL9*, plays an essential role in regulating miR399f expression and phosphate uptake in Arabidopsis, highlighting the intricate interplay between TFs and miRNAs in Pi homeostasis regulation.

Additionally, research has revealed complex Pi‐responsive regulatory networks centered around long non‐coding RNAs (lncRNAs) and miRNAs. For instance, lncRNAs, such as *At4* in Arabidopsis and *INDUCED BY PHOSPHATE STARVATION 1* (*IPS1*) in rice, are significantly upregulated under Pi deficiency. *IPS1* contains a sequence complementary to miR399, acting as a target mimic to sequester and inhibit the action of miR399 on *PHO2* (Figure 5) (Franco‐Zorrilla et al., 2007). Another lncRNA, *PI‐DEFICIENCY‐INDUCED LNCRNA 1* (*PILNCR1*), identified in maize RNA libraries, inhibits miRNA399‐guided cleavage of the *ZmPHO2* transcript, conferring Pi stress tolerance (Du et al., 2018). Moreover, *PILNCR2*, transcribed from the opposite strand of *ZmPHT1;1*, forms RNA/RNA duplexes with *ZmPHT1* mRNAs, blocking miR399‐guided cleavage. Overexpression of *PILNCR2* enhanced low‐Pi tolerance and increased *ZmPHT1;3* and *ZmPHT1;13* mRNA levels, while *PILNCR2* mutants showed reduced low‐Pi tolerance, underscoring its role in Pi homeostasis regulation in maize (Figure 5) (Wang et al., 2023c). Knockout of the miR399 target gene *ZmPHO2* in maize led to premature senescence. The floral transition regulator INDETERMINATE 1 (ID1) protein inhibits *ZmMIR399* transcription, reducing miR399's repression of *ZmPHO2* and aiding Pi homeostasis. ID1 regulates Pi independently of Pi status, with *ZmPHO2* exhibiting increased Pi sensitivity due to domestication (Wang et al., 2023b). In rapeseed, overexpression of Bna‐miR399c enhances Pi uptake, taproot growth, and biomass under low‐Pi stress by targeting *BnPHO2* (Du et al., 2023). In citrus, downregulation of CsmiR399a.1 disrupts floral development by altering Pi levels and gene expression. CsmiR399a.1 targets *UBIQUITIN‐CONJUGATING ENZYME 24* (*CsUBC24*), a homolog of Arabidopsis *PHO2*, which affects the regulatory network involving the SEPALLATA gene *CsSEP1* and the anther dehiscence regulator *INDUCER OF CBF EXPRESSION 1* (*CsICE1*), ultimately leading to developmental defects in flowers (Wang et al., 2020). These observations suggest that the miR399‐*PHO2* module acts as a conserved regulatory system of Pi homeostasis across diverse crops.

Other miRNAs also play significant roles in regulating Pi homeostasis in plants. Like miR399, miR827 translocates between roots and shoots during Pi starvation in Arabidopsis and rice (Huen et al., 2017; Lin et al., 2018). Unlike other miRNAs that target homologous genes across species, miR827 targets two distinct **S** YG1/**P** HO81/**X** PR1 (SPX) domain‐containing genes: *PHOSPHATE TRANSPORTER 5* (*PHT5*) and *NLA* to modulate Pi transport and storage in Arabidopsis and rice (Lin et al., 2018). In rice, osa‐miR827 negatively regulates **M**AJOR **F**ACILITY **S**UPERFAMILY genes, *OsSPX‐MFS1* and *OsSPX‐MFS2*, which are involved in Pi sensing or transport under varying external Pi conditions (Figure 5) (Wang et al., 2012). In Arabidopsis, miR827 targets *NLA*, which, in turn, regulates Pi homeostasis by suppressing Pi transport‐related genes, such as *PHT1* and *PHD FINGER PROTEIN 1* (*PHF1*), under N‐limited conditions, thereby preventing excessive Pi accumulation (Lin et al., 2013). In maize, overexpressing ZmmiR528 impairs root growth and Pi uptake, while its suppression enhances tolerance to low Pi by upregulating *ZmLac3* and *ZmLac5*, which are critical for root development. Hybrid maize plants with anti‐miR528 and *ZmLac3* overexpression showed improved root growth, Pi uptake, and resistance to Pi scarcity (Pei et al., 2024), demonstrating the potential for breeding programs.

Previous research has revealed complex stress‐responsive regulatory circuits where TFs and miRNAs act as key nodes in Pi homeostasis. In rice, the R2R3‐MYB transcription factor *OsMYB2P‐1* regulates osa‐miR399a and osa‐miR399j in response to Pi deprivation (Dai et al., 2012). Similarly, the *OsWRKY74* modulates the transcriptional activity of osa‐miR399a, osa‐miR399f, and osa‐miR399j under Pi ‐deficient conditions. Overexpression of *OsWRKY74* in rice enhances the expression of these miR399 family members (Dai et al., 2016). In Arabidopsis, miR156 and its target *SPL3*, a major regulator in Pi starvation response, directly modulate the expression of miR399f, *PHT1;5*, and *PHOSPHOLIPASE DZ 2* (*PLDZ2*) genes to enhance Pi uptake (Lei et al., 2016a). *SPL9*, another target of miR156, interacts directly with miR399f promoters at GTAC sites to regulate its expression. Overexpression of *SPL9* reduces anthocyanin accumulation under Pi stress, highlighting its role in Pi deficiency responses (Lei et al., 2023a). Furthermore, miR156 regulates genes involved in proton efflux and H^+^‐ATPase activity, enhancing rhizosphere acidification under Pi limitation. Overexpression of miR156 boosts acid secretion and root development, while its inhibition reduces H^+^‐ATPase activity and acidification (Lei et al., 2016b). Together, these findings underscore the critical functions of miRNAs in regulating Pi homeostasis in plants. Understanding these regulatory mechanisms provides a foundation for developing strategies to improve crop productivity in Pi‐limited environments.

#### miRNA gatekeepers of sulfur homeostasis

Sulfur, primarily existing as sulfate (SO_4_^2−^) in nature, is essential for plant growth and metabolism (Wawrzyńska and Sirko, 2024). Sulfate transporters (SULTRs) assist in sulfate transportation from roots to shoots and its translocation from mature leaves to developing tissues. Following sulfate absorption, it is activated by ATP sulfurylase and subsequently incorporated into sulfur‐containing metabolites (Rahimzadeh Karvansara et al., 2023). miRNAs, particularly the miR395 family in Arabidopsis, are critical regulators of sulfate transport and assimilation (Jagadeeswaran et al., 2014).

**Figure 6:**
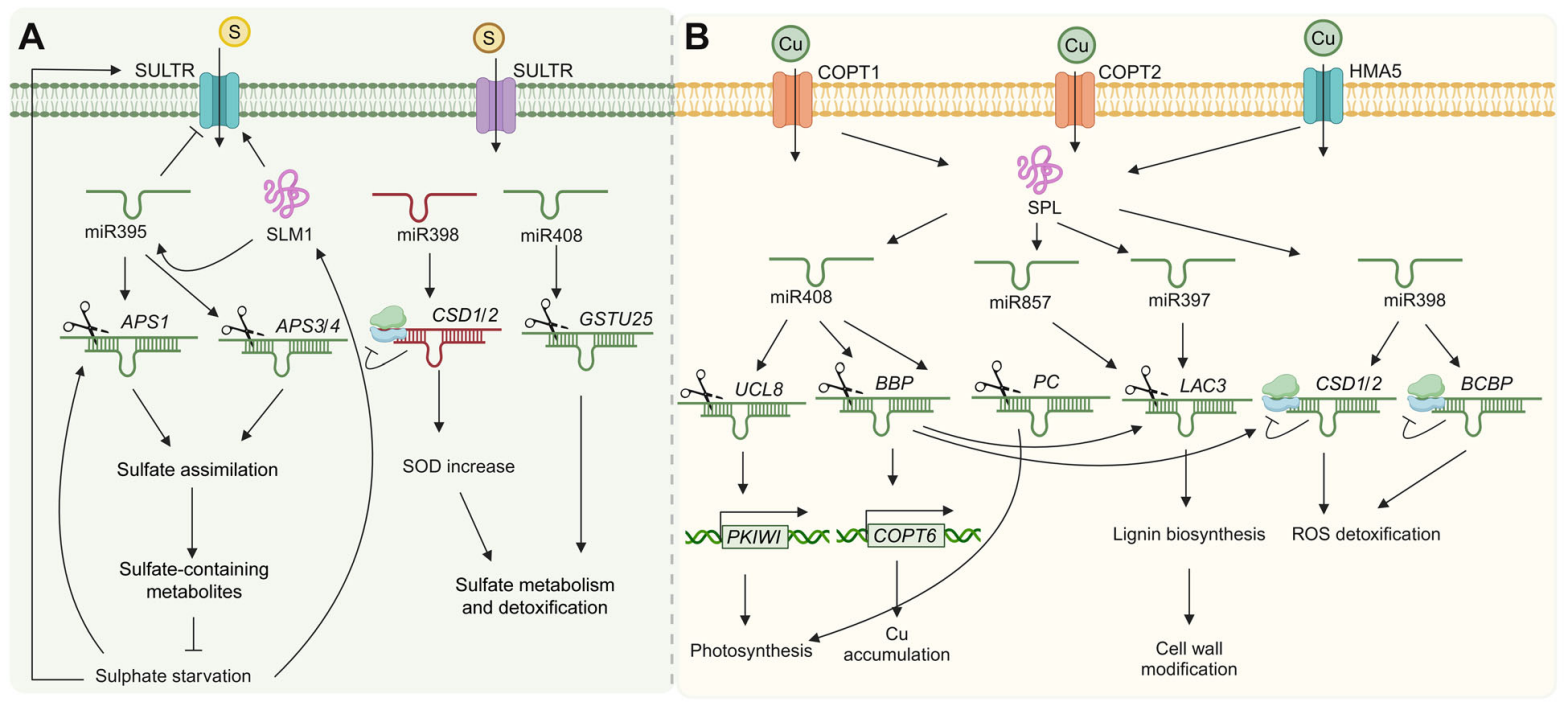
Overview of miRNA regulation in sulfur metabolism and response to copper stress in plants **(A)** In Arabidopsis, miR395 regulates sulfate assimilation and distribution by targeting ATP sulfurylase (APTS) and sulfate transporter genes (*SULTR2;1*), a process coordinated with the *SLIM1* transcription factor. In rice, miR395 enhances resistance to pathogens by suppressing *OsAPS1* and *OsSULTR2;1/2;2*, which promotes sulfate accumulation and pathogen resistance. Under sulfate stress in Arabidopsis, miR398 is downregulated, leading to the upregulation of *CSD1* and *CSD2*, which enhances SOD activity and provides protection against oxidative stress. Furthermore, miR408 regulates sulfur assimilation and stress responses in Arabidopsis by targeting *GSTU25*. Overexpression of miR408 or miPEP408 disrupts sulfur pathways, increasing sensitivity to stress, while miR408 mutants exhibit enhanced stress tolerance. **(B)** In rice, miR408 targets *OsUCL8*, facilitating Cu allocation for photosynthesis and reproductive development. Similarly, in Arabidopsis, miR408 targets *UCLACYANIN 2* and *PHOSPHATASE*, ensuring proper Cu allocation to essential proteins for photosynthesis and chloroplast development. Furthermore, miR398 targets *CSD1*, *CSD2*, and *CCS1*, regulating Cu homeostasis and stress responses during root‐knot nematode infection. In Arabidopsis and tomato, miR408, miR397, and miR857 target laccases and CSDs, regulating Cu homeostasis under Cu‐deficient conditions. Red color indicates downregulation of miRNAs, while green color indicates upregulation.

In Arabidopsis, miR395 regulates sulfate translocation and assimilation during sulfate starvation. Working alongside the *SULFUR LIMITATION 1* (*SLIM1*) transcription factor, miR395 modulates ATP sulfurylase (ATPS) transcript levels, increasing sulfate flux through the assimilation pathway under deficient conditions (Figure 6A) (Kawashima et al., 2011). While reduced ATPS expression alone impacts sulfate translocation and flux, the SULTR2;1 transporter is essential for efficient sulfate transport to the shoots, highlighting the complex role of miR395 in the regulatory circuit of plant sulfate assimilation (Matthewman et al., 2012). Furthermore, miR395 expression responds to metabolite treatments that modulate sulfate assimilation. Overexpression of miR395 represses *APS1*, *APS3*, *APS4*, and *SULTR2;1* under both normal and sulfate‐starved conditions, causing sulfate accumulation in the shoots and limiting redistribution from older to younger leaves (Figure 6A) (Liang et al., 2010; Kawashima et al., 2011). Interestingly, miR395 enhances resistance to bacterial pathogens *Xoc* and *Xoo* in rice by modulating sulfate accumulation and distribution. It suppresses the ATPS (*OsAPS1*) gene and sulfate transporter genes *OsSULTR2;1* and *OsSULTR2;2*, promoting sulfate accumulation and broad‐spectrum pathogen resistance (Figure 6A) (Yang et al., 2022c). Overexpressing OsamiR395h in tobacco impaired sulfate homeostasis by targeting the *NtaSULTR2* gene, which also responds to sulfate starvation (Yuan et al., 2016). In *Populus*, miR395c inhibits sulfate metabolism by targeting ATPS genes, reducing ABA synthesis and *MYB46* expression, which affects secondary xylem development (Liu et al., 2022).

Another key miRNA, miR398, is downregulated under sulfate stress, leading to increased transcript levels of its targets, *CSD1* and *CSD2* in Arabidopsis. This upregulation boosted SOD activity, protecting against sulfate‐induced oxidative stress (Figure 6A) (Li et al., 2017). A recent study in Arabidopsis identified miR408 as a key regulator of sulfur assimilation and stress responses, targeting *GSTU25*. Overexpression of miR408 or miPEP408 disrupts sulfur pathways and glutathione accumulation, increasing sensitivity to low sulfur and combined stresses. Conversely, miR408 mutants show enhanced tolerance to these conditions (Kumar et al., 2023). While most studies have focused on Arabidopsis, further investigation into miRNA roles in sulfur homeostasis across diverse plant species is needed to enhance crop performance under sulfur‐limited conditions. These insights may improve crop yields and promote sustainable agriculture in sulfur‐deficient soils.

#### miRNA managers of potassium metabolism

Plants adapt to low potassium (K^+^) levels by altering root growth and structure, inhibiting primary root elongation while promoting root hair development. Although miRNAs have been implicated in K^+^ deficiency responses, the direct mechanism by which they regulate K^+^ uptake remains unclear. However, certain studies have suggested that miRNAs influence K^+^ signal transduction.

For instance, in tomatoes, miR319 regulates low‐K^+^ stress resilience by targeting *SlTCP10*, which affects the expression of *JASMONIC ACID 2* (*SlJA2*). This pathway integrates ABA signaling to enhance low‐K^+^ tolerance. Overexpression of SlmiR319b delays root development and reduces K^+^ levels under stress conditions (Liu et al., 2023). In addition, miR168 modulates the sRNA regulatory pathway by targeting AGO1 in tomato plants. Overexpression of pri‐SlmiR168a improves root K^+^ content under deficiency conditions compared with WT and *SlAGO1A*‐resistant plants (Liu et al., 2020b), highlighting the role of miR168 in low‐K^+^ stress resilience.

In wheat, miR166d targets *TaCPK7‐D*, a calcium‐dependent protein kinase that regulates K^+^ uptake. The miR166d/*TaCPK7‐D* module modulates the expression of K^+^ transporters, *ARABIDOPSIS K* ^*+*^ *TRANSPORTER 1* (*TaAKT1*), and *HIGH‐AFFINITY K* ^*+*^ *TRANSPORTER 1* (*TaHAK1*), contributing to low‐K^+^ tolerance in wheat (Lei et al., 2023b). Similarly, tae‐miR408 is differentially expressed under K^+^ deprivation, targeting genes involved in secondary metabolism and ADP‐binding. Overexpression of tae‐miR408 in tobacco enhances K^+^ uptake, photosynthesis, and ROS scavenging, improving low‐K^+^ tolerance (Zhao et al., 2020).

Sugarcane roots under low‐K^+^ conditions exhibit differential expression of 36 miRNAs, including miR171‐x and miR156‐x/z, which regulate ethylene signaling and lateral root formation, respectively (Zhang et al., 2022c). Tibetan wild barley exhibits altered expression of 65 miRNAs under low‐K^+^ stress, such as miR164c, miR395a, and miR169h, which impact the TCA cycle and glycolysis. Genotype‐specific variations in miR160a, miR396c, and miR169h influence photosynthetic regulation under K^+^‐deficient conditions (Ye et al., 2021). In peanuts, K^+^ deficiency upregulates miRNAs such as miR156, miR390, miR160, miR164, and miR393, which are involved in pathways that help plants cope with both K^+^ and N scarcity (Li et al., 2021). In banana, miR160a targets *ARF10* and *ARF16* genes. Overexpression of miR160a in Arabidopsis impairs low‐K^+^ tolerance, resulting in shorter roots and reduced K^+^ accumulation (Chen et al., 2022). Thus, these findings underscore the intricate roles of miRNAs in modulating low‐K^+^ stress responses across various plant species. Although miRNAs regulate essential processes like K^+^ uptake, root development, and metabolic pathways, their underlying mechanisms require further investigation.

#### Iron shapes miRNAs in plants

Iron (Fe) is essential for all organisms as a cofactor in many enzymatic reactions. However, in calcareous soils with high pH and bicarbonate content, insoluble Fe becomes fixed, reducing its availability to plants. Recent research highlights the role of miRNAs in managing oxidative damage caused by Fe deficiency.

In Arabidopsis, miR164 emerges as a key regulator of Fe‐deficiency responses. Under Fe‐deficient conditions, *mir164b* mutants exhibited longer root growth, increased ferric reductase activity, and higher expression of *FERRIC REDUCTION OXIDASE 2* (*FRO2*) and *IRON‐REGULATED TRANSPORTER 1* (*IRT1*). *NAC5*, regulated by miR164, is upregulated under Fe‐deficient conditions, and its overexpression, along with *NF‐YA8*, increase tolerance by promoting root growth and Fe uptake (Du et al., 2022), highlighting the importance of the miR164b‐mediated *NAC5‐NF‐YA8* regulatory module in Fe‐deficiency responses.

Another important miRNA, miR408, exhibits the opposite regulation under Fe and copper deficiencies in Arabidopsis. It post‐transcriptionally controls several laccase‐like multicopper oxidase genes, such as *LAC3*, *LAC12*, and *LAC13*. Overexpression of miR408 impairs performance and reduces Fe‐regulated gene expression, while miR408 knockout plants maintain better phenol oxidase and ferroxidase activities under Fe deficiency (Carrió‐Seguí et al., 2019). In citrus, Fe deficiency triggers the differential expression of numerous miRNAs. Notably, reduced expression of miR172 enhances stress tolerance, while downregulation of miR395 contributes to sulfur homeostasis. The reduced expression of miR398 and miR408 is linked to improved Cu/Zn superoxide dismutase activity (Jin et al., 2021), contributing to better copper and Fe homeostasis.

In tomato roots, SlymiR157 enhances Fe‐deficiency responses by targeting the SPL transcription factor SlSPL‐CNR. This miRNA acts upstream of *SlbHLH101*, a key regulator of Fe uptake genes. SlSPL‐CNR interacts with specific motifs in the *SlbHLH101* promoter to repress its expression, thereby contributing to iron homeostasis (Zhu et al., 2022). Moreover, in transgenic rice seeds, Fe and zinc levels were significantly increased compared with WT seeds across the dough, milk, and mature stages. sRNA sequencing identified several miRNAs crucial for Fe uptake, such as miR399, miR166, and miR408. Novel miRNAs were found to activate the *NRAMP4* gene, a metal transporter, along with upregulation of *YELLOW STRIPE‐LIKE PROTEIN 15* (*OsYSL15*), *OsFRO2*, and *OsIRT1*, contributing to enhanced Fe transport and loading in transgenic rice seeds (Paul et al., 2016). Despite these findings, the functional significance of many miRNAs in Fe‐deficiency responses remains largely unexplored. Future research should focus on investigating the roles of miRNAs in Fe‐deficiency responses and their associated pathways. This knowledge could lead to the development of Fe‐efficient crop varieties, addressing issues in agriculture on calcareous soils.

#### miRNA‐mediated regulation of copper stress

Copper (Cu) uptake is crucial for plant physiology; however, even moderate imbalances in Cu levels can significantly impair photosynthetic pigments and disrupt the structure of chloroplasts and thylakoid membranes (Trentin et al., 2022; Wairich et al., 2022). miRNAs play a crucial role in regulating plant responses to fluctuating Cu levels, ensuring plant health and adaptability under Cu stress. Recent studies have emphasized the important role of miRNAs in modulating these responses.

In rice, miR408 regulates Cu homeostasis by targeting the *OsUCL8* (UCLACYAN) gene, which encodes a plantacyanin protein involved in pollen tube growth and fertility. This regulation enhances Cu allocation to essential proteins, such as plastocyanin, thereby supporting proper reproductive development. Additionally, *OsUCL8* interacts with *OsPKIWI*, a homolog of the Arabidopsis FNRL protein, potentially regulating *VITAMIN B1* (*VB1*) production, further highlighting the broader role of miR408 in regulating copper‐dependent processes (Zhang et al., 2018). In Arabidopsis, miR408, regulated by *SPL7* and *ELONGATED HYPOCOTYL 5* (*HY5*), maintains Cu homeostasis and mitigates light response issues by suppressing non‐essential Cu‐binding proteins, ensuring Cu is allocated to essential proteins like plastocyanin (Figure 6B) (Zhang et al., 2014). In apple, the miR408a‐*BBP‐LAC3/CSD1* module regulates Cu homeostasis and anthocyanin biosynthesis. Cu deficiency increases miR408a levels, which suppresses *BASIC BLUE PROTEIN* (*BBP*), limits Cu‐dependent enzymes, and upregulates *COPPER TRANSPORTER 6* (*COPT6*), promoting Cu accumulation and anthocyanin production. Excess Cu reduces miR408a expression, increasing *BBP* levels, and elevating *CSD1* and *LAC3* levels, which sequester excess Cu and reduce ROS‐induced stress (Figure 6B) (Hu et al., 2023b; Hong et al., 2024). These studies highlight the conserved role of miR408 in regulating Cu homeostasis across various plant species.

The miR398 family, along with miR408, is regulated by the Cu‐responsive *SPL7*, which modulates Cu homeostasis and stress response in Arabidopsis and tomato. Under Cu deficiency, miR408 suppresses *UCLACYANIN 2* (*UCC2*) and *PHOSPHATASE 2G* (*PP2CG1*), while miR398 reduces *BLUE COPPER BINDING PROTEIN* (*BCBP*), *CSD1*, and *CSD2*, balancing Cu distribution and stress response (Figure 6B) (Noureddine et al., 2022). Similarly, in hickory, cca‐miR398 targets *CSD1*, *CSD2*, and *CSD3*, reducing antioxidant enzyme activity and Cu tolerance, which impairs root growth and detoxification under Cu and sulfate stress (Figure 6B) (Sun et al., 2020).

Another miRNA, miR397, also plays a significant expression in Cu stress responses in plants. For instance, in banana, miR397 is upregulated under Cu deficiency, targeting *Musa‐LAC8* and *Musa‐LAC11* to reduce multicopper oxidase activity, thereby conserving Cu for essential processes like photosynthesis by regulating *Musa‐COPT* and *Musa‐FRO2* expression (Patel et al., 2019). In Arabidopsis, miR397sa, miR398b/c, and miR857, regulated by the transcription factor *SPL7*, target laccases, and CSDs to conserve Cu during stress (Gielen et al., 2016). Conversely, miR156, influencing SPL transcription factors like *SPL3*, competes with *SPL7* for binding to Cu‐miRNA promoters (Perea‐García et al., 2021), integrating Cu homeostasis with developmental and environmental responses.

In grapevine, suppression of miRNAs (miR408, miR398, and miR397) results in the upregulation of their target genes encoding Cu‐containing proteins. Experimental validation by RLM‐RACE confirms the negative regulation of *vvi‐LAC4* and *vvi‐LAC17* by miR397 and *vvi‐PC* and *vvi‐LAC12* by miR408, thereby controlling Cu availability (Figure 6B) (Leng et al., 2017). Similarly, a group of miRNAs differentially expressed under Cu stress, including miR156, miR172, miR169, miR398, miR408, and miR399, has been identified in grapevine. These miRNAs target various transcription factors and Cu‐related genes, enabling grapevines to adapt to Cu stress while maintaining essential physiological processes (Jiu et al., 2019). These studies elucidate the regulatory pathways and vital roles of miRNAs in Cu homeostasis, providing insight into how plants respond to Cu. However, future research should focus on understanding complex regulatory pathways, investigating crosstalk between Cu and other metals, and determining plant‐specific miRNA regulatory mechanisms to advance our knowledge of how plants adapt to Cu stress.

#### Boron stress alters miRNA signaling

Boron (B) is a crucial micronutrient for plants, with a narrow range between deficiency and toxicity. Recent research has highlighted the significant role of miRNAs in managing B stress by regulating gene expression across various plant species (Suresh Kumar et al., 2023). In Arabidopsis and citrus, miR397 emerges as a key player in B stress management by regulating the expression of laccase‐like proteins involved in cell wall lignification. Under high‐B conditions, miR397 targets *CsiLAC4* and *CsiLAC17* for mRNA cleavage, thereby downregulating their expression (Huang et al., 2022). Conversely, in orange, miR397 decreases under excess B conditions, leading to the upregulation of its target gene *LAC7*, which increases lignin biosynthesis (Jin et al., 2016), forming a barrier that minimizes B uptake and enhances B toxicity tolerance.

Citrus species show differential responses to B toxicity. In B‐toxic *Citrus grandis* leaves, miR395a and miR397a are significantly upregulated, while they are downregulated in *Citrus sinensis* leaves (Huang et al., 2016). Additionally, in *C. sinensis*, other miRNAs, including miR474, miR394, miR782, miR843, and miR5023 target specific genes involved in ROS inactivation, lateral root formation, and nutrient uptake, collectively contributing to the plant's adaptive response to B stress (Lu et al., 2014). Similarly, *Citrus* roots exposed to B toxicity show differential miRNA expression, with distinct roles: miR319 suppresses lateral root formation to minimize B uptake, while miR171 targets SCL genes to maintain root elongation. Additionally, miR396g‐5p modulates ion transport, collectively supporting the plant's adaptation to B stress (Huang et al., 2019).

Cotton plants overexpressing miR408 exhibit enhanced tolerance to B stress, maintaining water balance and cell integrity by upregulation of boron transporters genes, *BORON TRANSPORTER 1* (*BOR1*) and *BOR2*, as well as aquaporins, *PLASMA MEMBRANE INTRINSIC PROTEIN 1;1* (*PIP1;1*) and (*PIP2;1*) (Erkan and Akcay, 2024). In barley, B treatment induces tissue‐specific changes in miRNA expression, with miR408 and miR5180 showing notable upregulation, potentially regulating signal transduction pathways and demonstrating the complex, tissue‐specific miRNA responses to B stress in different plant species (Ozhuner et al., 2013). Together, these studies provide a deeper understanding of plant responses to B stress, highlighting the complex interplay between miRNAs and their target genes in managing B homeostasis.

#### Manganese stress induces regulatory miRNAs

Manganese (Mn) is an essential micronutrient for plants, but excessive Mn in acidic or waterlogged soils can negatively affect plant growth and development. Although several Mn transporter families have been identified, there is limited understanding of how miRNAs regulate these transporters to maintain Mn homeostasis in plants (Ju et al., 2022).

In Arabidopsis, Mn toxicity triggers a complex miRNA‐mediated regulatory network. The downregulation of miR395 and miR399a disrupts sulfur assimilation and phosphate homeostasis by targeting ATPS genes, *SULTR2;1*, and *UBC24/PHO2*. Upregulation of miR826 influences glucosinolate biosynthesis by targeting *METHYL ESTERASE 7* (*MES7*) and *MES9*, while increased expression of miR5595 and miR5995b modulates the systemic response to Mn stress through methyl esterase genes (Gong et al., 2019). In common beans under Mn stress, miRNAs regulate development across tissues: miR319 targets TCP factors in roots and nodules, miR399 targets *GERMIN‐LIKE PROTEIN 1* (*GRL1*) in leaves and roots, miR170 targets SCL genes in nodules, and miR156 targets SPL factors across tissues (Valdés‐López et al., 2010). These studies highlight the complex and tissue‐specific nature of miRNA‐mediated regulation in plant responses to Mn stress. However, our understanding of Mn stress responses in crops remains limited, necessitating the need for further research to develop effective strategies for mitigating the adverse effects of excessive Mn on plant growth and development.

In summary, current research on miRNAs in plant nutrient stress responses has primarily focused on identifying downstream target genes; however, our understanding of upstream regulatory elements controlling miRNA expression remains limited, especially in the context of rhizosphere dynamics. Accumulative evidence has shown that nutrient stress triggers significant changes in miRNA expression profiles, which are crucial components of gene regulatory networks that govern plant responses to nutrient fluctuations. For instance, miR399 plays a pivotal role across Pi, K^+^, Mn, and Fe stresses by regulating *PHO2*, which is essential for phosphorus homeostasis and nutrient translocation between roots and shoots. Similarly, miR164, involved in N, Fe, and K^+^, stress responses, modulates NAC transcription factors, impacting nutrient uptake and root development. The multifaceted roles of these miRNAs underscore the complexity and interconnection of plant responses to different nutrient stresses. Future research should focus on elucidating the mechanisms by which upstream elements modulate miRNA expression, particularly nutrient uptake and translocation from the rhizosphere. This approach could enhance our understanding of plant biology and offer strategies to improve food production and security. Targeting specific miRNAs or their regulators may help enhance plant nutrient use efficiency and stress resilience, contributing to sustainable agriculture in changing environments.

### miRNAs: fine tuning plant responses to salinity

Soil salinization is a major factor influencing the plant rhizosphere, significantly affecting crop yield, quality, and overall production. Plants respond to salt stress through various mechanisms, including modifications at the transcriptional, post‐transcriptional, translational, and post‐translational levels (Van Zelm et al., 2020). Among these mechanisms, miRNAs play a crucial role by regulating target genes involved in salt stress responses.

Several miRNAs regulate hormone pathways under salt stress, particularly the auxin response pathway. miRNAs, such as miR160 and miR390, target ARFs, modulating auxin signaling to enhance salt tolerance. For instance, in poplar, overexpression of miR390 increases tasiRNA production, which represses *ARF4* and boosts salt tolerance, while miR390 knockdown inhibits root growth under salt stress (He et al., 2018). Similarly, in cotton, silencing of ghr‐miR160b increases salt sensitivity, while increasing miR160b expression or inhibiting the expression of its target genes, *GhARF17/18*, promotes root development and salt resistance (Zhang et al., 2021). In soybean, gma‐miR4359b enhances salt‐stress tolerance by negatively regulating the expression of its target, F‐box protein *GmFBX193* (Yu et al., 2023). The reduced miR167 expression in transgenic tobacco upregulates *AUXIN RESISTANT 1* (*AUX1*), *PIN‐FORMED 1* (*PIN1*), and *PIN2*, boosting salt‐stress tolerance. Additionally, the coordinated expression of miR156, miR159, and miR394, and their target genes, including *SPL9*, *ARF6*, and *ARF8*, highlights their role in modulating auxin signaling and enhancing salt tolerance across various plant species (Makkar et al., 2022).

miRNAs are also involved in other hormone pathways, such as ABA and ethylene metabolism, which are crucial for salt‐stress responses. For instance, miR172c positively regulates salt tolerance in soybean and Arabidopsis by enhancing ABA sensitivity (Li WenBin et al., 2016; Sahito et al., 2017). In contrast, overexpression of osa‐miR171c in rice reduces salt resistance and increases sensitivity to ABA (Yang et al., 2017). Another key player, miR399f, enhances salt tolerance in Arabidopsis by downregulating the expression of its predicted target genes, *CHLOROPLAST STEM‐LOOP BINDING PROTEIN 41* (*CSP41b*) and *ABF3*, both involved in ABA signaling (Baek et al., 2016). In response to salt stress, TaemiR408 enhances salt tolerance by upregulating ABA receptor gene *PYRABACTIN RESISTANCE 1‐LIKE 2* (*NtPYL2*) and the SnRK2 gene *STRESS‐ACTIVATED PROTEIN KINASE 3* (*NtSAPK3*), thereby promoting osmolyte accumulation and improving plant growth (Bai et al., 2018). Similarly, miR319 increases salt tolerance in *creeping bentgrass* by inhibiting *PvPCF5*, a TCP transcription factor, which leads to ethylene accumulation and modulates stress‐responsive genes (Liu et al., 2019).

miRNAs play a crucial role in regulating various TFs involved in salt‐stress response. miR172 suppresses the expression of AP2‐like transcription factors, such as *SALT SUPPRESSED AP2 DOMAIN‐CONTAINING 1* (*SSAC1*) in soybean, thereby enhancing salt tolerance (Pan et al., 2016). Similarly, miR156 targets SPL transcription factors, with varying effects across species. For instance, in apple, overexpression of miR156 reduces salt resistance, while overexpression of its target gene, *MdSPL13*, enhances salt tolerance. The miR156‐SPL module can upregulate *MdWRKY100* to regulate the salt tolerance in apple (Ma et al., 2021). Additionally, overexpression of rice‐derived miR396c in *creeping bentgrass* enhances salt tolerance by targeting growth‐regulating transcription factors, specifically downregulating *AsGRF3‐6* to improve salt resistance (Yuan et al., 2019). A study by Yue et al. (2020) demonstrated that inhibiting or knocking out OsmiR535, along with its target *OsSPL19*, significantly enhanced rice tolerance to salinity by increasing the number of lateral roots, primary root length, and shoot length (Yue et al., 2020). However, the mechanisms by which miRNAs target transcription factors during salt stress, including whether these factors are activated or repressed, remain poorly understood.

Salt stress induces oxidative stress through the accumulation of ROS, and miRNAs play a significant role in regulating ROS metabolism and maintaining cellular redox balance. A recent study in rice and wheat showed that miR172a/b is highly expressed in salt‐treated plants, where it negatively regulates its target gene, the AP2/ERF domain‐containing transcription factor *INDETERMINATE SPIKELET 1* (*IDS1*), which is crucial for responding to ROS production (Cheng et al., 2021). Similarly, in rice, miR12477 targets L‐ASCORBATE OXIDASE (*LAO*), an enzyme responsible for ascorbate breakdown, thereby limiting ROS accumulation under salt stress (Parmar et al., 2020). In cotton, miR414c regulates ROS homeostasis by repressing *GhFSD1*, which encodes iron superoxide dismutase. Knockdown of *GhFSD1* leads to excessive ROS buildup and increased salinity stress sensitivity (Wang et al., 2019b). Additionally, in tomato, miR398b regulates copper/zinc superoxide dismutase, contributing to ROS removal and improving salt tolerance (He et al., 2021).

Maintaining ion homeostasis is a critical component of salt‐stress adaptation, and miRNAs play a significant role in regulating this process. In poplar, miR319a enhances salt tolerance by modifying xylem structure and upregulating the genes *HIGH‐AFFINITY K* ^*+*^ *TRANSPORTER 1* (*PagHKT1;2*) and *STELAR K* ^*+*^ *OUTWARD RECTIFIER 1* (*PagSKOR1‐b*), which are involved in Na^+^ efflux and K^+^ influx. These changes improve ion transport and contribute to salt‐stress tolerance (Cheng et al., 2024). In birch, miR408a targets *BpBCP1*, a blue copper protein, modulating antioxidant activity and Na^+^ homeostasis. Interestingly, overexpression of miR408a increases stress sensitivity, while overexpression of *BpBCP1* enhances salt tolerance by improving antioxidant responses and maintaining ion balance (Liu et al., 2024b). Moreover, in sorghum, miR‐6225‐5p inhibits the expression of the Ca^2+^ uptake gene *GLUTAMATE RECEPTOR‐LIKE 3* (*SbGLR3.1*), reducing calcium levels in the root epidermis and affecting the plant's ability to withstand salt stress (Sun et al., 2023). Thus, miRNAs are integral to plant responses to salt stress, regulating hormonal pathways, transcription factors, ROS metabolism, and ion homeostasis. Understanding the role of miRNAs in salt stress is essential for gaining a deeper insight into the mechanisms of gene regulation.

## CONSERVED MIRNA REGULATORS IN THE RHIZOSPHERE: INTEGRATING RESPONSES TO ABIOTIC AND BIOTIC STRESS

Next‐generation sequencing has greatly expanded the availability of plant genome and transcriptome data, facilitating the identification of conserved miRNAs across species and revealing their evolutionary patterns (Cui et al., 2017). While certain miRNA families are conserved across land plants and angiosperms, others are species specific or limited to closely related plants (Sunkar et al., 2012; Megha et al., 2018). These miRNAs, along with their target genes, form complex regulatory networks that play a crucial role in coordinating plant stress responses (Hausser and Zavolan, 2014).

In plants, the conservation of miRNA biogenesis and processing machinery (You et al., 2017) is complemented by the remarkable conservation of certain mature miRNA gene families across evolutionarily distant yet related species (Bartel, 2004; Axtell, 2013). For example, miRNAs such as miR160, miR167, and miR393 regulate TIR1 and ARF genes, modulating the responses to nutrients, salt, and heavy metals. These miRNAs are also induced during bacterial infection, highlighting the convergence of biotic and abiotic stress signals. The differential regulation of auxin signaling by miRNAs appears to be a common strategy employed by plants to coordinate stress responses. miR398 targets members of the SOD family, including *CSD1* and *CSD2*, as well as *COX5b.1*, modulating oxidative stress responses and nutrient homeostasis. Apart from its differential regulation in response to abiotic stress, its downregulation affects *CSD1* but not *CSD2* during pathogen infection. Similarly, miR171 targets SCL proteins, influencing root formation, hormone signaling, and pathogen resistance. miR395, miR399, and miR408 regulate sulfur assimilation, Pi, and Cu homeostasis, as well as salt‐stress responses, while also contributing to pathogen defense.

Many miRNAs target TFs crucial for plant growth and stress responses (Pagano et al., 2021). For instance, miR156 targets the SPL family, influencing root architecture and responses to nutrients and pathogens. Interestingly, miR156 is also downregulated when plants are challenged by fungal pathogens, suggesting a role in suppressing plant immunity against certain bacterial pathogens. Other miRNA targeting TFs include miR319, which targets TCP, miR169, which targets NF‐YA, and miR159, which targets MYB, to influence root development and defense against pathogens and nematodes. Additionally, miR164 targets NAC transcription factors, playing a role in nutrient uptake, root development, and responses to abiotic stress. While under biotic stress, the upregulation of miR164 miRNA has been observed in cotton in response to *V. dahliae*.

In conclusion, these conserved miRNAs highlight the intricate connections between plant responses to nutrients, heavy metals, and salt‐stress responses, as well as pathogen defense in plants, underscoring the complexity of regulatory networks that govern plant adaptation to rhizosphere challenges. Investigating these stress‐responsive miRNAs across different tissues will be crucial for unraveling tissue‐specific regulatory mechanisms (Li et al., 2022). Therefore, by understanding these shared functions, we can develop targeted strategies to manipulate miRNA‐mediated gene regulation, ultimately improving crop resilience and productivity under stress conditions.

## MIRNA HIJACKING IN CROSS‐KINGDOM RHIZOSPHERIC SIGNALING

As previously discussed, various signaling molecules are exchanged in the rhizosphere. Many microbial signaling molecules involved in communication with host plants also participate in antimicrobial communication (Sasse et al., 2018). Interestingly, miRNAs can move within plants and across kingdoms, influencing gene expression in distant organisms. Their stability and cross‐kingdom transfer are likely to have been enhanced by ribose methylation, association with RNA‐binding proteins, and packaging into exosomes (Zhao et al., 2012). Most examples of miRNA cross‐kingdom transfer occur in plant–parasite or plant–pathogen interactions, where they induce gene silencing (Gualtieri et al., 2020), a phenomenon known as cross‐kingdom RNA interference (RNAi) (Cai et al., 2018b).

Moreover, the export of silencing RNAs by hosts to suppress the expression of virulence genes in pathogens represents a fascinating defense mechanism against fungal infections. For instance, *Botrytis cinerea* sRNAs (Bc‐sRNAs) can hijack the host RNAi mechanism in Arabidopsis and tomato by binding to AGO1 and silencing immunity‐related genes. These fungal sRNAs act as effectors, inhibiting host immunity, and depend on the DCL1 and DCL2 proteins of *B. cinerea*. The *dcl1/dcl2* double mutant, unable to produce these sRNA effectors, exhibited significantly reduced pathogenicity (Zotti et al., 2018). Since then, sRNAs, particularly miRNAs, have emerged as key mediators of these interactions, sparking increasing research interest. For example, in *V. dahlia‐*infected cotton, the production of miR166 and miR159 increases, leading to cleavage of transcripts of *CA* ^*2+*^ *‐DEPENDENT CYSTEINE PROTEASE* (*Clp‐1*) and *ISOTRICHODERMIN C‐15 HYDROXYLASE* (*HiC‐15*) genes, which are essential for hyphal growth and microsclerotium formation, respectively (Zhang et al., 2016a). Similarly, a trans‐kingdom fungal sRNA, VdrsR‐1, derived from *V. dahliae*, retards host plant floral transition by targeting the miR157d precursor *MIR157d*. This sRNA increases miR157d accumulation in infected plants rather than reducing it. Through this mechanism, VdrsR‐1 disrupts the regulatory role of the miR157 family, which is known for its involvement in SPL gene regulation across plant species. Consequently, *SPL13A/B* expression is significantly reduced in *V. dahliae*‐infected and VdrsR‐1‐expressing plants, leading to delayed vegetative phase change and floral transition (Zhang et al., 2022a). In tomato, the novel miRNA miR1001 inhibited the virulence of *B. cinerea*‐infected plants, with miR1001/miR1001* showing a stronger effect than miR1001 alone. Additionally, miR1001 significantly inhibits conidiospore germination of *B. cinerea* by targeting *Bcin10g01400.1* and *Bcin03g02170.1* genes, which encode ATP‐dependent metallopeptidase and cysteine‐type endopeptidase, respectively (Meng et al., 2020).

Recently, increasing numbers of studies have highlighted the role of miRNA exchanges in nonpathogenic relationships, particularly focusing on host–microbiota interactions in the gut (Middleton et al., 2021). Remarkably, ingested plants release extracellular vesicles (EVs) in the gut, delivering miRNAs to target bacteria along with plant miRNAs, altering gene expression, and influencing metabolite production, thereby affecting gut microbiota (Teng et al., 2018). Plant miRNAs, often tissue‐specific, are abundant in roots, and a subset may be excreted via EVs into the rhizosphere, where they coevolve with surrounding organisms (Breakfield et al., 2012). Based on recent advances in understanding the impact of host and plant miRNAs on the gut microbiota (Middleton et al., 2021), we propose that plants and their rhizospheric microbiota also communicate via miRNAs, regulating microbial composition and activity in the rhizosphere. Therefore, further research is necessary to elucidate the role of miRNAs in plant–microbe communication, particularly the influence of plant miRNAs on rhizospheric microbial communities.

## CHALLENGES AND LIMITATIONS OF MIRNA TRANSCRIPTOME IN THE RHIZOSPHERE

The increasing availability of genomic data from diverse plant species has advanced ncRNA discovery, yet the accurate prediction of ncRNAs remains challenging. Identifying miRNA targets is often described as “a classical needle in a haystack problem” (Arias and Busskamp, 2019). Understanding the regulatory roles of miRNAs in plant–rhizosphere networks requires identifying and validating miRNA–target interactions. However, experimentally validating numerous potential target sites is time‐consuming and costly (Zhao et al., 2021).

Over the last two decades, research into miRNAs has expanded significantly, leading to a surge in annotated miRNAs in plants and animals. Despite similarities in transcription by RNA polymerase II, miRNA genes possess distinct features (Xie et al., 2005). In plants, miRNA precursors are generally more variable in length and often form complex secondary structures, such as atypical stem loops and bifurcate‐branch loops (Kuang et al., 2022). This variability, along with their longer precursors compared with animal miRNAs, complicates their annotation (Moro et al., 2018). Another challenge in predicting and analyzing miRNAs is the presence of multiple members within conserved families, often resulting from whole genome and tandem duplication events (Baldrich et al., 2022).

Although significant progress has been made in plant miRNA annotation, the rhizosphere remains understudied due to its inherent complexity. Initially, miRNA identification relied on sequence alignment and molecular experiments like northern blotting (Liu et al., 2020a). Real‐time qPCR offers high sensitivity, but its application is limited by the availability of spectrally resolved fluorescent probes (Xu et al., 2022b). Similarly, planar microarrays, which can detect thousands of miRNAs, face challenges due to low sensitivity. The emergence of NGS techniques revolutionized plant miRNA annotation by uncovering numerous non‐conserved or species‐specific miRNAs (Sunkar and Zhu, 2004). This was further complemented by the bioinformatics tools that analyze NGS sRNA libraries to identify miRNA features, significantly enhancing the annotation process (Zhao et al., 2021). Despite these advances, bioinformatics tools often struggle to filter out noise from other small RNAs, such as siRNAs, and even updated annotation criteria that emphasize distinctions between miRNAs and other sRNAs (Axtell and Meyers, 2018). These challenges are also prevalent in plant miRNA databases, which frequently suffer from low signal‐to‐noise ratios (SNR) and a lack of standardized annotation criteria (Raabe et al., 2014). Understanding miRNA conservation across plant genomes is particularly challenging due to their short sequences, high copy numbers, unique expression patterns, diverse precursors, and the difficulty of designing specific probes for detection and quantification (Rajesh et al., 2021). While miRNAs hold potential as biomarkers, their application in the complex rhizosphere environment presents additional hurdles (Mahadevakumar and Sridhar, 2020). Functional analysis of miRNAs using technologies such as TALENs and CRISPR‐Cas9 faces several challenges, including the selection of effective gRNAs, system delivery, and minimizing off‐target mutations (Abbas et al., 2022). Moreover, miRNA breeding processes are lengthy, and enhancing traits must not compromise the desirable original characteristics. Identifying gaps in stress‐induced miRNA production may provide valuable insight into rhizosphere cues. To advance our understanding of miRNA roles in the complex rhizosphere environment, improved computational tools and novel methods to bridge laboratory and field research will be essential.

## CONCLUDING REMARKS AND FUTURE PROSPECTIVES

Since the discovery of miRNAs, significant progress has been made in understanding their biogenesis and the functions of this versatile class of repressors. In addition to their well established roles in plant development and stress responses, future research should focus on exploring their role in plant–microbe interactions and abiotic stress in the rhizosphere. miRNAs play essential roles in regulating plant growth, development, and responses to rhizosphere signals, with plants evolving complex miRNA systems to adapt to various rhizosphere factors (Song et al., 2019).

Manipulating miRNAs is more challenging than manipulating mRNAs, requiring sophisticated techniques and a comprehensive understanding of their biogenesis and functions to enable effective interventions. Due to the short length of miRNAs, engineering tools with significantly higher sensitivity and resolution are needed to accurately target specific regions for miRNA gene manipulation (Rojas et al., 2020). Given the substantial contributions of miRNAs to various aspects of plant life, it is crucial to develop tools for manipulating miRNAs in conserved lineages and across kingdoms, as they play important roles in plant adaptation and rhizosphere interactions (Yu et al., 2017). In recent decades, versatile miRNA engineering tools have been developed to target both transcriptional and post‐transcriptional levels. As miRNA manipulation holds the potential for enhancing crop productivity, quality, and tolerance to rhizosphere stresses, genome editing has become the preferred method for improving agriculturally important plants, ensuring stable inheritance of desired traits across generations. Research on miRNA‐target genes has revealed a greater level of complexity than initially expected, highlighting the need for continued efforts to understand miRNA–target interactions. miRNAs show potential as early biomarkers for assessing the effects of various rhizospheric biotic and abiotic cues. In addition, it is crucial to explore how miRNAs influence the complex interactions between plants, pathogenic microbes, and nutrient imbalances in the rhizosphere, and how these factors collectively impact plant health and disease. Understanding these dynamics could provide valuable insight for improving agricultural practices and enhancing crop resilience in the belowground environment, while maintaining optimal nutrient homeostasis in crops. Special focus should be directed toward genome editing, target expression modification, and artificial target generation to enhance desirable traits in crops (Deng et al., 2022). This knowledge is critical for advancing breeding and genetic engineering strategies.

The secondary structure of miRNAs plays a critical role at the intermediary level, containing essential information due to its significant folding energy. These structures are evolutionarily conserved and often offer explanations for observed experimental findings (Andrews, 2021). In addition, it may be intriguing to explore whether the identity of mismatch pairs, particularly C–C mismatches, impacts other biological processes (Rojas et al., 2020). To advance miRNA analysis, innovative methods for predicting miRNA localization are needed, as they could facilitate the discovery of new pre‐miRNA species. Furthermore, investigating how rhizosphere factors (such as microbial signals, heavy metal stress, and nutrient fluctuations) affect plant miRNA secondary structures could provide valuable insight into their stability, target recognition, and potential for cross‐kingdom movement.

The advent of NGS technology for single‐cell sequencing (scRNA‐seq) has unveiled the internal mechanisms operating within individual cells. However, in recent years, only limited numbers of studies have delved into the regulatory mechanisms of miRNAs at the single‐cell level in both plants and animals (Brosnan et al., 2019). As scRNA‐seq technology matures, it is expected that the molecular mechanisms of plant miRNAs will be analyzed at the cellular level (Yang et al., 2023). Therefore, scRNA‐seq of miRNAs could reveal complex physiological, structural, and functional relationships within individual cells across tissues, particularly during plant–microbe interactions. Unlike scRNA‐seq, spatial RNA sequencing preserves cellular positions within tissues, enabling us to map miRNA expression patterns across plant structures and revealing how miRNA‐mediated signals propagate in response to rhizosphere cues. Future advancements in scRNA‐seq and spatial RNA sequencing technologies are likely to enhance our ability to discover novel miRNAs and deepen our understanding of the functions of known miRNAs in the plant rhizosphere. These improvements will provide increased sequencing throughput and spatial resolution of RNA activity, offering comprehensive insight into miRNA biology.

In summary, based on advances in miRNA manipulation and genetic engineering, future successes in miRNA regulation are highly anticipated. Expanding genome editing to enable the precise control of miRNA genes will accelerate plant biology research and address pressing agricultural challenges. However, further research is needed to explore the role of miRNAs in plant–microbe interactions, particularly their impact on rhizospheric microbial communities. These advancements could enhance crop breeding, improve adaptation to environmental changes, and contribute to global food security amid rapid population growth.

## CONFLICTS OF INTEREST

The authors declare no conflict of interest.

## AUTHOR CONTRIBUTIONS

L.W. conceived the review topic and designed the writing. M.F. wrote the major manuscript and prepared the main figures and tables. L.T. and W.L. contributed to writing sections of the text and figure preparation. L.W. revised the manuscript. All authors read and approved the final manuscript.

## Supporting information

Additional Supporting Information may be found online in the supporting information tab for this article: http://onlinelibrary.wiley.com/doi/10.1111/jipb.13860/suppinfo

**Table S1.** List of miRNAs involved in plant–rhizosphere biotic interactions
**Table S2.** List of heavy metal‐stress‐responsive miRNAs in the plant rhizosphere
**Table S3.** List of nutrient‐stress‐responsive miRNAs in the plant rhizosphere
