# Supplementary material for: MicroRNA gatekeepers: Orchestrating rhizospheric dynamics
Source: J Integr Plant Biol. 2025 Feb 21;67(3):845–76. doi: 10.1111/jipb.13860 (PMC11951408; doi:10.1111/jipb.13860)
Supplement: Supplementary file 1 — Table S1. List of miRNAs involved in plant–rhizosphere biotic interactions Table S2. List of heavy metal‐stress‐responsive miRNAs in the plant rhizosphere Table S3. List of nutrient‐stress‐responsive miRNAs in the plant rhizosphere [file JIPB-67-845-s001.docx]

**Table S1. List of miRNAs involved in plant rhizosphere biotic interactions.**

| Microbes | Crop | miRNA | Mechanism |  | Target gene/mRNA | Causal agent | Reference |
| --- | --- | --- | --- | --- | --- | --- | --- |
| Bacteria | Arabidopsis | miR393 | Enhances Arabidopsis resistance |  | *AFB2, AFB3, TIR1* and *LecRLK* | pstDC3000 | (Navarro et al., 2006; Djami-Tchatchou & Dubery, 2015) |
|  | Arabidopsis | miR393b* | Secrete pathogenesis-related protein PR1 |  | *MEMB12* | pstDC3000 | (Zhang et al., 2011) |
|  | Arabidopsis | miR156 | Suppresses plant immunity |  | *SPL9* | pstDC3000 | (Yin et al., 2019) |
|  | Arabidopsis | miR825* | Increase susceptibility |  | *TIR-NBS-LRR type R genes* | pstDC3000 | (Niu et al., 2016) |
|  | Arabidopsis | miR398 | Decreased resistance |  | *CSD1, CSD2 and COX5b-1* | pstDC3000 avrRpm1  pstDC3000 avrRpt2 | (Niu et al., 2016) |
|  | Arabidopsis | miR164c | Increase susceptibility |  | *P5CS1* |  | (Jagadeeswaran et al., 2009) |
| Fungus | Arabidopsis | miR858 | Negative regulator of immunity |  | *flavonoid-specific MYB* | Plectosphaerella cucumerina, Fusarium oxysporum and Colletotrichum higginsianum | (Li et al., 2010) |
|  | Arabidopsis | miR773 | Increased susceptibility |  | *MET2* | Plectosphaerella cucumerina, Fusarium oxysporum and Colletotrichum higginsianum | Gupta et al., 2020 |
|  | Arabidopsis | miR159  miR166 | Increase virulence by interfering with nuclear export of the AGO1–miRNA complex |  | ALY family proteins | *Verticillium dahliae* | (Pérez-Quintero et al., 2012) |
|  | Cotton | miR530 | Attenuated plant resistance |  | *SAP6* | *V. dahliae* | (Camargo-Ramírez et al., 2018) |
|  | Cotton | miR165  miR395 | Sulfur assimilation |  | *REV*  *APS1/3* | *V. dahliae* | (Soto-Suárez et al., 2017) |
| Nematode | Arabidopsis and tomato | miR408 | Decrease resistance |  |  | *Meloidogyne incognita* | (Salvador-Guirao et al., 2018) |
|  |  | miR398 |  |  |  |  |  |
|  | Arabidopsis | miR159 | Increased susceptibility |  | *MYB33* | *Meloidogyne incognita* | (Zhu et al., 2022) |
|  | Arabidopsis | miR396 | Reduces susceptibility by inhibiting cell proliferation |  | *GRF* | root-knot nematode | (Hu et al., 2023) |
|  |  | miR319 | Enhances resistance through JA signaling |  | *TCP4* |  |  |
|  | Cotton | miR319  miR159 | Increased susceptibility |  | *TCP4*  *MYB* | root-knot nematode | (Mei et al., 2022) |
|  |  | miR167 | Decreased susceptibility |  | *ARF8* |  |  |
|  | Arabidopsis | miRNA172 | Increased resistance |  | *TOE1* | Meloidogyne javanica | (Hu et al., 2020) |
|  | Arabidopsis | miR827 | Increased susceptibility |  | *NLA* | *Heterodera schachtii* | (Noureddine et al., 2022) |
|  | Soybean | miR4407 | Inhibit nodulation |  | *GmIPT3* |  | (Medina et al., 2017) |
| Root nodule | *M. truncatula* | miR169 | nodule development |  | *NF-YA1* | *Sinorhizobium meliloti* | (Combier et al., 2006; Zanetti et al., 2020) |
|  | Soybean  *Lotus japonicus* | miR2111 | Promotes nodulation |  | *TML* | *Bradyrhizobium diazoefficiens,*  *Mesorhizobium loti* | (Zhang et al., 2021; Okuma et al., 2020) |
|  | Soybean | miR4407 | Reduces number of nodules |  | *GmIPT3* | *Sinorhizobium fredii* | (Fan et al., 2023) |
|  | Soybean | miR172c | Increases nodule formation |  | *GmNNC1* | *Bradyrhizobium diazoefficiens,*  *Bradyrhizobium japonicum* | (Wang et al., 2019; Wang et al., 2014) |
|  | Soybean | miR160 | Enhances cytokinin and auxin sensitivity |  |  | *Bradyrhizobium japonicum* | (Nizampatnam et al., 2015) |
|  | Soybean | miR167 | Positively regulates nodule formation |  | *ARF8* | *Bradyrhizobium japonicum* | (Wang et al., 2015) |
|  | Soybean  *M. truncatula* | miR393 | Reduce nodule number |  | *TIR1* | *Bradyrhizobium japonicum* | (Cai et al., 2017) |

**Table S2. List of heavy metal-stress-responsive miRNAs in the plant rhizosphere.**

| Stress | Plant specie | miRNA | Target gene/mRNA | Associated pathway | References |
| --- | --- | --- | --- | --- | --- |
| Cd | *Arabidopsis* | miR397 | *LAC2/4/17* | Lignin biosynthesis | (Ali et al., 2023) |
|  |  | miR398 | *CSD1, SOD* | ROS regulation | (Yan et al., 2023) |
|  |  | miR156 | *AtHMA4* | Cd transportation | (Zhang et al., 2020) |
|  | Rice | miR268 | *NRAMP3* | Development and ROS | (Ding et al., 2017) |
|  |  | miR166 | *HB4* | Cd accumulation | (Ding et al., 2018) |
|  |  | mir535 | *Nramp5* | Cd uptake and accumulation | (Yue et al., 2023) |
|  | *B. napus* | miR167 | *NRAMP1b* | Metal transportation | (Meng et al., 2017) |
|  | Barley | miR156g-3p_3 | *NAT2* | Stress signaling, glutathione metabolism, and phytochelatin synthesis | (Wang et al., 2023) |
|  | Tabacco | miR156 | *SPL4a* | Cd tolerance | (He et al., 2023) |
|  | Wheat | miR398 | *CSD* | Cd stress signaling | (Qiu et al., 2016) |
|  |  | IamiR-4-3p | *GST3, AWPM19-like* | Cd accumulation | (Shen et al., 2022) |
|  | Maize | miR18642–3p miR171k-5p miR21151–3p, miR30996–3p | *ABC transporters,*  *peroxisomes,*  *GSH metabolism,*  *ubiquitin-proteasome* | Cd accumulation | (Teng et al., 2023) |
| Al | Barley | miR166b | *HOX9* | cell wall Al binding and H+ influx | (Feng et al., 2020) |
|  |  | miR393 | *TIR1, AFB* | regulates root sensitivity to Al through auxin signaling | (Bai et al., 2017) |
|  |  | miR319 | *TCP4* |  | (Wu et al., 2018) |
|  |  | miR160 |  |  |  |
|  |  | PC-miR1 |  | cell wall pectin biosynthesis |  |
|  |  | miR393 |  |  |  |
|  | Soybean | gma-miR396c/k, gma-miR166k/o, and gma-miR390g, |  | root elongation | (Huang et al., 2018) |
|  |  | gma-miR169r |  | oxidative stress |  |
|  | *Phaseolus vulgaris* | MIR1511 | *ALS3* | Al sensitivity | (Ángel Martín‐Rodríguez et al., 2021) |
|  | Common bean | miR393 | *TIR1* | Gibberellin and auxin signaling | (Mendoza-Soto et al., 2015) |
|  |  | miR164 | *NAC1* |  |  |
|  |  | miR170 | *SCL* |  |  |
|  | *Medicago truncatula* | miR156g-3p |  | Root cell growth | (Lu et al., 2023) |
|  |  | novel_miR_36,  novel_miR_182,  novel_miR_135 | *bHLH, MYB, GRAS, WRKY* | Al-tolerance regulation |  |
|  | *Vitis quinquangularis* | miR172b, miR477b-3p |  | Al-tolerance regulation | (Jiang et al., 2023) |
|  | Flax | miR393, miR390, miR319 |  | Regulation of plant growth | (Dmitriev et al., 2017) |
| Arsenic | *Brassica juncea* | miR156, miR169, miR172 |  | Plant development | (Srivastava et al., 2013) |
|  |  | miR395, miR838, miR854 |  | Metal assimilation |  |
|  |  | miR319, miR167, miR164, miR159 |  | Hormonal signaling pathways |  |
|  | Maize | miR156s, miR166e-3p, miR166m, miR319b-3p |  | plant growth and development | (Ghosh et al., 2017) |
|  |  | miR159b-5p.3, zma_460,  zma_468, zma_469 |  | metabolic processes |  |
|  |  | miR167d |  | ROS generation |  |
|  |  | miR167d, miR159e.2, miR319b-3p |  | hormone signaling pathways |  |
|  | Rice | miR156j | *LecRLKs, UGT, WAKs, ZFPs* | lipid metabolic process | (Pandey et al., 2020) |
|  |  | miR528 |  | ROS regulation | (Liu et al., 2015; |
|  |  | miR396, miR399, miR408, miR528, miR1861, miR2102, miR2907, miR164, miR171, miR395, miR529, miR820, miR1432, miR1846 |  | arsenic detoxification, uptake, transport, and stress tolerance | Sharma et al., 2015) |
|  | Arabidopsis | miR408 | *GSTU25* | Glutathione and sulfur pathways | (Kumar et al., 2023) |
| Cr | Radish | miR156, miR159, miR160, miR168, miR169, miR319, miR397, miR398, miR399, miR408, miR161, miR172, miR390, miR394 | *Heavy metal ATPase (HMA), yellow stripe-like 1 transporter (YSL1),* and *ATP-binding cassette (ABC) transporter proteins* | Cr uptake and homeostasis | (Liu et al., 2015) |
|  | Maize | miR156a, miR164, miR396d, miR155 | *cytochrome P450 gene,* NAC transcription factors, mitogen-activated protein kinase and MT protein gene | Physiological, biochemical metabolisms and Cr detoxification | (Adhikari et al., 2023) |
|  |  | miR159c | *GAMYB* | Plant growth |  |
|  | Rice | miR159 | *MAPK* cascade | MAPK activity and auxin signaling | (Dubey et al., 2020) |

**Table S3. List of nutrient-stress-responsive miRNAs in the plant rhizosphere.**

| Stress | Plant specie | miRNA | Target gene/mRNA | Associated pathway | References |
| --- | --- | --- | --- | --- | --- |
| N | Arabidopsis | miR167 | *ARFs* | Plant development and nitrate regulation | (Gutierrez et al., 2012; Jagadhesan et al., 2022) |
|  | Arabidopsis, Soybean | miR393 | *TIR1, AFBs* | Auxin signaling and nitrate regulation | (Bao et al., 2014; Guan, 2017) |
|  | Rice | miR444 | *MADS27, ANR1* | Root growth and nitrate regulation | (Shin et al., 2018; Pachamuthu et al., 2022) |
|  | Sugarcane | miR168 | *SPX* | NUE regulation | (Gao et al., 2022) |
|  |  | miR396 | *acnA* |  |  |
|  | Arabidopsis  Rice | miR169 | *NFYA* | NUE regulation | (Liang et al., 2012; Xu et al., 2014; Seo et al., 2023) |
|  | Potato | miR397  miR398 |  |  | (Tiwari et al., 2020) |
| P | Arabidopsis | miR399 | *PHO2* | Regulation of phosphate uptake and translocation | (Liu et al., 2014; Pei et al., 2024) |
|  | Rapeseed |  |  |  | (Du et al., 2023) |
|  | Maize |  | *PHTs* |  | (Y. Wang et al., 2023) |
|  | Citrus |  | *UBC24* | Development as well as regulation of phosphate uptake and translocation | (Wang et al., 2020) |
|  | Rice | miR827 | *SPX-MFS1 and SPX-MFS2* | Phosphate (Pi) sensing or transport | (Wang et al., 2012) |
|  | Arabidopsis |  | *NLA* | Regulation of P homeostasis | (Lin et al., 2013) |
|  | Maize | miR528 | *LAC3* and *LAC5* | Growth and regulation of P homeostasis | (Pei et al., 2024). |
|  | Arabidopsis | miR156 | *SPL3* | Regulation of P homeostasis | (Lei et al., 2016) |
| S | Arabidopsis | miR395 | *APS1, APS3,* and *APS4* | Regulation of sulfate assimilation pathway | (Kawashima et al., 2011; Ai et al., 2016) |
|  | Tobacco |  | *SULTR2* | Sulphate homeostasis | (Liu et al., 2022) |
|  | Arabidopsis | miR398 | *CSD1 and CSD2* | Regulation of S homeostasis | (Li et al., 2017) |
|  | Arabidopsis | miR408 | *GSTU25* | Regulation of S pathways and glutathione accumulation | (Kumar et al., 2023) |
| K^+^ | Tomato | miR319 | *SlTCP10* | Modulate abscisic acid (ABA) signaling under K^+^ fluctuation | (Liu et al., 2023) |
|  | Wheat | miR166d | *TaCPK7-D* | Regulation of K^+^ homeostasis | (Lei et al., 2023) |
|  |  | miR168 | *SlAGO1A* | Regulation of development and K^+^ homeostasis | (Liu et al., 2020) |
|  | Sugarcane | miR171-x, miR156-x/z |  | Ethylene signaling and root growth | (Zhang et al., 2022) |
|  | Barley | miR164c, miR169h, and miR395a |  | (TCA) cycle, glycolysis, and pentose phosphate pathways |  |
|  |  | miR160a, miR396c, and miR169h |  | photosynthetic regulation | (Ye et al., 2021) |
|  | Arabidopsis | miR160a | *ARF10, ARF16* | Auxin signaling and K^+^ regulation | (Chen et al., 2022) |
|  | Tobacco | tae-miR408 |  | Metabolic pathways and ROS scavenging | (Zhao et al., 2020) |
| Fe | Arabidopsis | miR164 | *IRT1, FRO2, NAC5* | Regulation of Fe homeostasis and plant growth | (Du et al., 2022) |
|  |  | miR408 | *LAC3, LAC12, LAC13* | Lignin biosynthesis and regulation of Fe homeostasis | (Carrió-Seguí et al., 2019) |
|  | Tomato | miR157 | *SlSPL-CNR* | Fe homeostasis | (Zhu et al., 2022) |
|  | Rice | miR11, miR30, miR26, miR31 | *NRAMP4* | Regulation of iron transport | (Paul et al., 2016) |
|  | Citrus | miR172  miR395  miR398 miR408 |  | Regulation of iron transport | (Jin et al., 2021) |
| Cu | Malus | miR408 | *BBP* | copper (Cu) homeostasis and anthocyanin biosynthesis | (Hu et al., 2023) |
|  | Rice |  | *UCL8* | Regulation of Cu allocation to essential proteins | (Zhang et al., 2018) |
|  | Arabidopsis |  | *UCC2, PP2CG1* | Cu regulation and light signaling | (Zhang et al., 2014) |
|  | Arabidopsis and Tomato |  |  |  | (Noureddine et al., 2022) |
|  |  | miR398 | *CSD1, CSD2, and BCBP* | Regulate copper homeostasis |  |
|  | *Carya cathayensis* |  | *CSD1, CSD2, and CSD3* | Regulate copper homeostasis | (Sun et al., 2020) |
|  | Banana | miR397 | *LAC8, LAC11* | Photosynthesis regulation | (Patel et al., 2019) |
|  | Grapevine | miR397  miR408 | *LAC4 and LAC17*  *PC and LAC12* | Control Cu availability | (Leng et al., 2017) |
| B | Arabidopsis and Citrus | miR397 | *LAC4* and *LAC17* | Lignin biosynthesis | (Huang et al., 2022) |
|  | *Poncirus trifoliata* |  | *LAC7* |  | (Jin et al., 2016) |
|  | *C. grandis* | miR395a miR397a | *LAC4, LAC7* |  | (Huang et al., 2016) |
|  |  | miR474 | *PDH* | optimizes ROS inactivation and development | (Lu et al., 2014) |
|  |  | miR394 | *LCR* |  |  |
|  |  | miR782 | *PDIL5-1 and MYBML2* |  |  |
|  |  | miR843 | *LRR* |  |  |
|  |  | miR5023 | *PPIase and RHD3* |  |  |
|  |  | miR830 | *kinesin motor-related protein* | nutrient uptake and transport |  |
|  |  | miR5266 | *ammonium transporter 2* |  |  |
|  |  | miR3465 | *Ca2+-ATPase 11* |  |  |
|  | Barley | miR408 | *heterotrimeric G protein alpha subunit and ATPase family gene 1* | signal transduction pathways | (Ozhuner et al., 2013) |
|  |  | miR171 | *SCARECROW-like* | stem cell differentiation |  |
| Mn | Arabidopsis | miR395 | *APSs, SULTR2;1* | nutrient transport and assimilation | (Gong et al., 2019) |
|  |  | miR399 | *UBC24/PHO2* | nutrient transport |  |
|  |  | miR826 | *AOP1, AOP2, and AOP3* | glucosinolate biosynthesis |  |
|  |  | miR5595 and miR5995b | *MES7 and MES9* | Regulation of systemic acquired  resistance and stress response |  |
|  | *Phaseolus vulgaris* | miR319 | *TCP, GRL1* | influencing root architecture and nutrient homeostasis | (Huang et al., 2019) |
|  |  | miR170 | *SCL* |  |  |
|  |  | miR156 | *SPL* |  |  |
|  |  | miR172 | *AP2* |  |  |
